# Sex Change in Clownfish: Molecular Insights from Transcriptome Analysis

**DOI:** 10.1038/srep35461

**Published:** 2016-10-17

**Authors:** Laura Casas, Fran Saborido-Rey, Taewoo Ryu, Craig Michell, Timothy Ravasi, Xabier Irigoien

**Affiliations:** 1 King Abdullah University of Science and Technology (KAUST), Division of Biological and Environmental Science & Engineering, Red Sea Research Center, Thuwal 23955-6900, Saudi Arabia; 2 Institute of Marine Research (IIM-CSIC), 36208 Vigo, Spain; 3 KAUST Environmental Epigenetic Program (KEEP), Division of Biological and Environmental Sciences & Engineering, Division of Applied Mathematics and Computer Sciences, King Abdullah University of Science and Technology, Thuwal 23955-6900, Kingdom of Saudi Arabia

## Abstract

Sequential hermaphroditism is a unique reproductive strategy among teleosts that is displayed mainly in fish species living in the coral reef environment. The reproductive biology of hermaphrodites has long been intriguing; however, very little is known about the molecular pathways underlying their sex change. Here, we provide the first *de novo* transcriptome analyses of a hermaphrodite teleost´s undergoing sex change in its natural environment. Our study has examined relative gene expression across multiple groups—rather than just two contrasting conditions— and has allowed us to explore the differential expression patterns throughout the whole process. Our analysis has highlighted the rapid and complex genomic response of the brain associated with sex change, which is subsequently transmitted to the gonads, identifying a large number of candidate genes, some well-known and some novel, involved in the process. The present study provides strong evidence of the importance of the sex steroidogenic machinery during sex change in clownfish, with the aromatase gene playing a central role, both in the brain and the gonad. This work constitutes the first genome-wide study in a social sex-changing species and provides insights into the genetic mechanism governing social sex change and gonadal restructuring in protandrous hermaphrodites.

Teleost fishes display the largest array of sex-determining systems among animals, resulting in a large number of reproductive strategies, a key factor in explaining their success during evolution^1^. Among these, functional hermaphroditism is a unique strategy^2^ that has been adopted by at least 27 families across seven orders of teleosts, mainly in the coral reef environment^3^. In *simultaneous* hermaphroditism individuals possess fully functional male and female gonads while in *sequential* hermaphroditism fish change sex sometime during its life, either from male to female (protandry) or from female to male (protogyny). Factors triggering sex change differ among species. In some, particularly in protandrous species, sex change is size dependent and eventually every fish in the population will change sex. In territorial-haremic species sex change is socially mediated, and it is more common in protogynous species. One interesting exception are the clownfishes (subfamily Amphiprioninae) which are protandrous, monogamous and sex change seems to be controlled socially^4^,^5^, i.e. male does not change sex when attaining a certain size, but only after the female disappearance. It has been suggested that sequential hermaphroditism in reef habitats improves adaptation, increases survival rates and enhances reproduction^3^. However, our understanding of the molecular pathways underlying reproductive processes, particularly sex change in hermaphrodites, is very limited.

Clownfishes (subfamily Amphiprioninae) are extensively distributed in tropical waters, where they inhabit shallow waters across the Red Sea, the Indian and the western Pacific Oceans^6^. They live in an obligate symbiosis with certain sea anemones that provide the fish with nesting sites and protection from predators^7^. The sessile nature of anemones makes clownfishes a good system for investigating socially-controlled sex change since the process can be monitored in experiments conducted in the field.

Clownfishes live in social assemblages as pairs or social groups consisting of a dominant female, always the largest in size, surrounded by a male and a variable number of immature juveniles of smaller size^8^. Clownfish species display a strong social hierarchy based on size^9^. These hierarchies function as queues for breeding. In a given group, the fish age and grow larger together, with their relative size differences and the dominance hierarchies among group members remaining unchanged^10^. Although a simplification, we hereafter call these social assemblages families. If the dominant female of a family dies, all subordinates seize the opportunity to ascend in rank and grow. The male is poised to become female and rapidly changes sex to assume the vacated position, while the largest undifferentiated fish completes the breeding pair by turning into a mature male in a short time^4^,^8^. This ability allows the formation of a new breeding pair, preventing the need for dangerous travel across the reef, but requires the presence of subdominant fish to complete the sex change^5^.

Shortly after the female is removed, the behavioral repertoire switches. The male who used to receive orders from the female now displays aggression and dominance, beginning to court the smaller fish as the female would. The brain mediates these behavioral changes^11^, although very little is known about the neural and transcriptional mechanisms that orchestrate sex change in fishes. These changes in the relative neuronal activity in the male fish’s brain are transmitted along the hypothalamic–pituitary-gonadal axis^12^. Receptors on the gonadal tissue receive the hormonal signals and resorb or extend accordingly, completing the gonadal sex change^13^. This process involves a complete reorganization of the gonadal tissue. The gonad of the functional males is an ovotestis, with the presence of both testicular and ovarian tissues^14^. However, while the testes are mature, the ovary is in an immature phase (with only oogonia and primary growth oocytes). At the time male begins to change sex it enters the transition phase^15^, which is characterized by the progressive degeneration of the testes concomitant with the proliferation of the ovarian tissue. At the end of the transitional phase the testicular tissue is resorbed and shortly after females become mature. The importance of gonadal sex steroid hormones, especially estrogens, as key regulators of sex change in clownfish has been proposed^16^. However, the upstream mechanisms controlling the production and activity of gonadal steroid hormones during sex change in clownfish remain largely unknown^1^.

Here, we aim to provide insights into the genetic mechanism governing social sex change in fish, using the Red Sea endemic species of clownfish, *Amphiprion bicinctus*, as a model in its natural habitat. We seek to shed light on the main gene regulatory networks involved in the whole sex change process by observing the cascade of expression changes, their specific patterns and their temporal autocorrelations via a transcriptomic approach. Our time-series expression experiment provides information regarding the complete set of genes that is activated, as well as the dynamics and interactions between these genes. Our analysis is focused on the two main organs involved in sexual development and reproduction, the brain and gonads^17^.

## Results

### Histological analysis of gonads indicates a gradual decrease of testicular tissue during the male-to-female sex change

Gonads were histologically analyzed in twelve sex-changing individuals, two functional males and six functional females that were used as controls for the completion of sex change (Table 1). The gonads of functional males had 55% female tissue, on average similar in value to the gonads of changing males collected on days 5 and 11. As sex change progressed, the gonads of changing males had a steadily decreasing amount of testicular tissue and increasing amounts of ovarian tissue, reaching 87% 40 days after the removal of the original females. During the last steps of the transition (days 40–50 after removal of the original females), individuals possessed highly degenerated and reduced testes, but their oocytes, although increased in size, remained in the primary-growth stage; i.e., none showed evidence of ripening (onset of vitellogenesis). All functional females were mature with vitellogenic oocytes in spawning-capable phase. Interestingly, one fish, CF10M, who belonged to a three-member family when it was originally tagged but was left alone in the anemone when the female was removed since the third member had disappeared, did not undergo the expected male-to-female sex change. The histological exam of CF10M’S gonads at the time of collection, 35 days after the original female was removed, did not show any signs of sex change and was therefore grouped in the male category.

For the correlation analysis, the index of sex change of each individual as measured by the percentage of female tissue in the gonad was used. For the pairwise differential expression analysis, individuals were grouped into five categories according to the percentage of female tissue in the gonad (Table 1): “male” (M), “transitional male” (TM), “transitional female” (TF), “immature female” (FI) and “mature female” (FM). Since the proportion of female tissue in functional males was 55% and 100% in functional females (45% difference), a group was defined every 15% increase in female tissue resulting in three transitional groups. One of these groups (TF) has only two individuals, while it should ideally have three or more, which may impair the power for detecting differences in expression. However, this risk of impairment is reduced due to the statistical approach taken and the fact that the selection criteria of the genes is based not solely on pairwise, but also on correlation analysis.

### Read pre-processing, de novo transcriptome assembly and abundance estimation

The sequencing process produced a total of 278 million paired-end reads (see Supplementary Table S1 for more detailed information). The selected assembly produced an estimated number of 516,599 Trinity transcripts, with a median contig length of 603 bp, mean of 1,025 bp and 43.58% GC content. Evaluation of the genome for completeness identified 95.6% complete and 100% partial genes from the 248 core eukaryotic genes dataset (CEGs). Analysis of the reconstructed transcripts using TransDecoder revealed a total of 96,589 contigs potentially containing candidate protein-coding regions. CD-HIT-EST was then used to merge similar contigs (identity threshold of 0.9) resulting in 47,065 contigs with an N50 of 1,563 bp.

### Differential Expression Analysis of brain tissue reveals a marked down-regulation during transitional stages and very little differences between mature males and females

Comparisons were conducted separately for the brain and gonadal samples, since the hierarchical clustering performed with the sample-to-sample distances revealed two well-differentiated groups (Supplementary Fig. S1A). Clustering analysis of each of the tissues separately shows that expression profiles from the brain samples display higher similarity than those from the gonad samples (Supplementary Fig. S1B,C).

**Figure 1:**
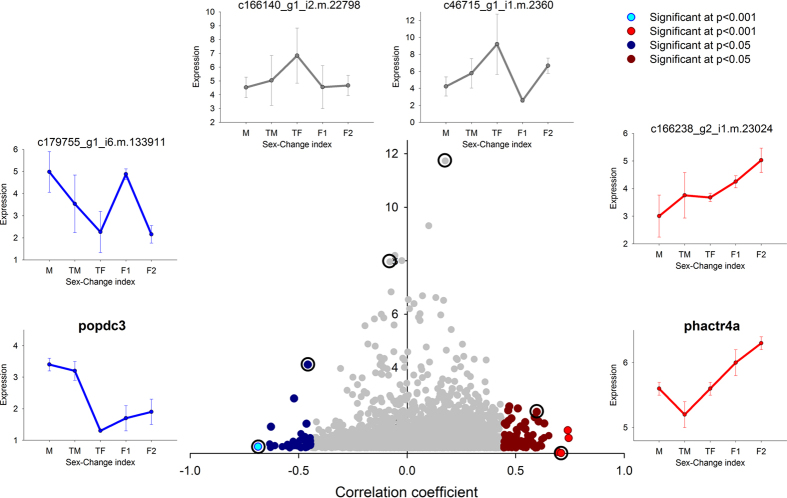
Changes in the gene expression profile of the brain during the sex change of clownfish (*Amphiphrion bicinctus*). Contigs are plotted by their correlation with the index of sex-change (x-axis) and the dispersion in expression among individuals (y-axis, only values above 1 are plotted). Significant correlations are highlighted in blue for negative and in red for positive coefficients, indicating up-regulation in males and females, respectively. Examples from selected contigs (marked with a circle) regarding its position in the plot are highlighted. Labels: M - males; TM – transitional males; TF – transitional females; FI – immature females; and FM – mature females.

To unveil the time course of the brain transcriptome throughout the sex change, we calculated the Pearson correlation coefficients between normalized expression levels in brain samples (DESeq_normalized) and the index of sex change of each individual. A total of 1,531 contigs showed significant correlation (p < 0.05) to the index of sex change, with 884 contigs positively correlated and 647 contigs negatively correlated. Only 12 contigs showed high significance (p < 0.001), eight with positive and four with negative correlation (see Supplementary Table S2). Therefore, more contigs were found to be female-biased than male-biased in the brain. The scatterplot of the correlations against the dispersion in expression shows that the distribution of correlation coefficients was similar on both sides of the spectrum, female and male, with largest values of 0.8, indicating that there was as much up-regulation at the beginning as at the end of the sex-change process (Fig. 1). Significantly correlated contigs displayed small dispersion, especially the contigs up-regulated at the end of the sex-change process, i.e., when the correlation was positive. The largest differences in expression were observed in contigs not correlated to the index of sex change (grey dots in Fig. 1), indicating that most of the differences in the brain samples were observed during transitional stages.

The analysis of differential expression among the five sex categories based on histology results revealed a significance (p < 0.001) in 163 contigs of which only two also showed correlation with the index of sex change (Supplementary Table S2). The vast majority of the differentially expressed contigs (DECs) were found in comparisons involving transitional stages (TM and TF), while very few contigs were differentially expressed between males (M) and female stages (FI, FM) (Table 2). In the brain, the main changes in expression occurred therefore during transitional stages, an observation that also explains the paucity of correlated contigs found in the previous analysis.

### Differential Expression Analysis of gonad tissue reveals a later response than in brain and large differences in expression between mature males and females

**Figure 2:**
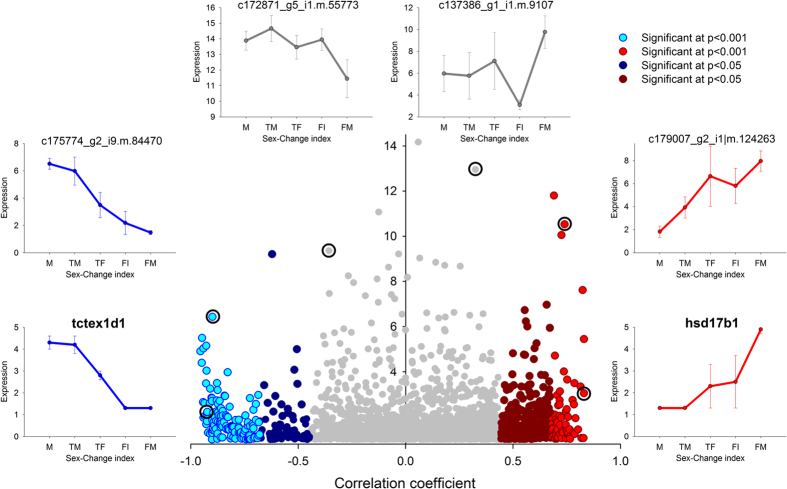
Changes in gonadal expression during the male-to-female sex change of clownfish (*Amphiphrion bicinctus*). Contigs are plotted by their correlation with the index of sex change (x-axis) and the dispersion in expression among individuals (y-axis, only values above 1 are plotted). Significant correlations are highlighted in blue for negative and in red for positive coefficients, indicating up-regulation in males and females, respectively. Examples from selected contigs (marked with a circle) regarding its position in the plot are highlighted. Labels: M - males; TM – transitional males; TF – transitional females; FI – immature females; and FM – mature females.

The Pearson correlation coefficients between normalized expression levels in gonad (DESeq_normalized) and the index of sex change of each individual revealed a total of 5,483 contigs showing significant correlation (p < 0.05), with 2,602 contigs positively correlated and 2,881 contigs negatively correlated. We found 558 contigs with highly significant correlations (p < 0.001); 206 of these were positively correlated, while the remaining 352 were negatively correlated (Supplementary Table S3). Thus, more contigs were found to be male-biased, the opposite trend than in the brain samples. The scatterplot of the correlations against the dispersion in expression (Fig. 2) shows that significantly correlated contigs displayed a wide range of differential expression. Significant positive correlations were generally smaller; the largest value was 0.8. Eighty-three negatively correlated contigs had coefficients above 0.8. Also the contigs that were up-regulated in females showed a larger range in differential expression. Strong correlation indicates a steady increase or decrease in expression, but often with small differences among individuals (Fig. 2). On the contrary there were contigs with large differences in expression, some of them not correlated with the index of sex change at the individual level (grey dots in Fig. 2), but displaying large differences in expression in some of the sex change categories. The plot of the dispersion for the gonads was similar to that for the brain although generally there were larger differences among individuals.

The multiple pairwise comparisons among the five sex categories at the gonadal level revealed a total of 434 contigs showing significant differential expression (p < 0.001), with 224 of them also showing correlation to the index of sex change (Supplementary Table S3). The majority (160) were negatively correlated while 64 were positively correlated. The majority of the significant DECs were found in comparisons involving mature females (Table 2), indicating that expression patterns during the last phase of the sex change are clearly distinct from all the previous stages. The largest number of DECs was found between male categories (M and TM) compared to mature females, and the smallest numbers between consecutive stages as expected (M-TM, TM-TF and TF-FI) (Table 2).

### PCA and cluster analyses of differential expression unravels the changes originating in the brain and subsequently transmitted to the gonads

**Figure 3:**
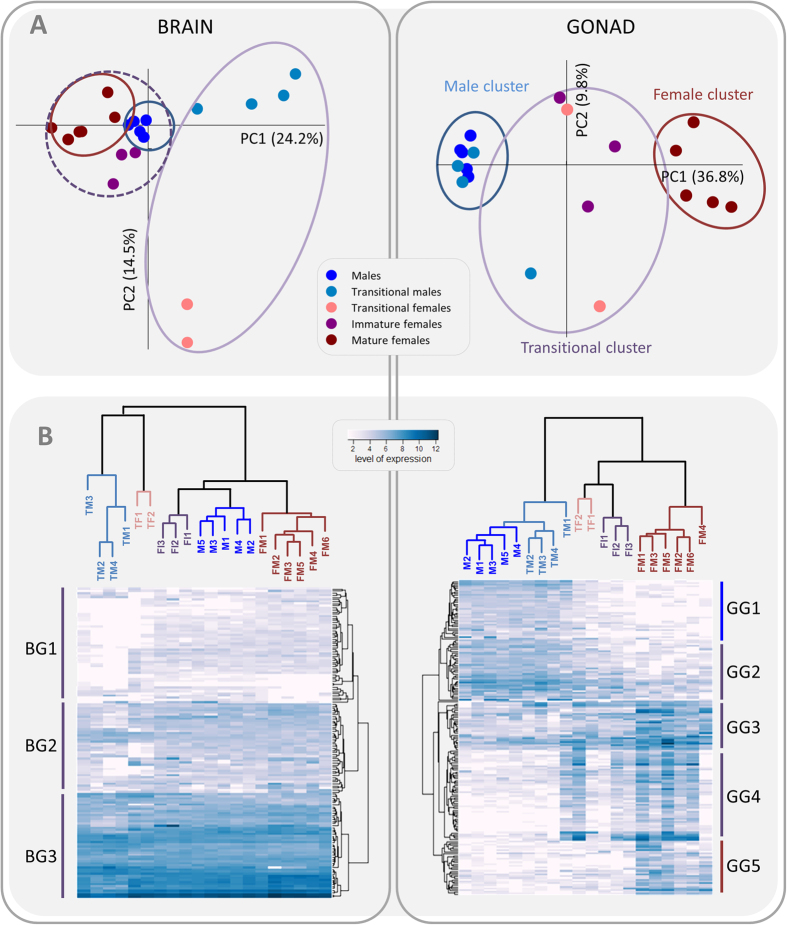
Multivariate analyses of RNA-Seq data in clownfish (*Amphiphrion bicinctus*). Gene expression changes were investigated by (**A**) Principal Component Analyses performed on normalized RNA-Seq data of a selected set of 173 transcripts from the brain (left) and 768 transcripts from the gonad (right). (**B**) Hierarchical dendrogram clustering on the same data. The clustering of transcripts results in three (brain: BG1 to BG3) and five (gonad: GG1 to GG5) clearly differentiated expression profiles. Labels: M – males (dark blue); TM – transitional males (light blue); TF – transitional females; FI (pink) – immature females (purple); and FM – mature females (brown).

**Figure 4:**
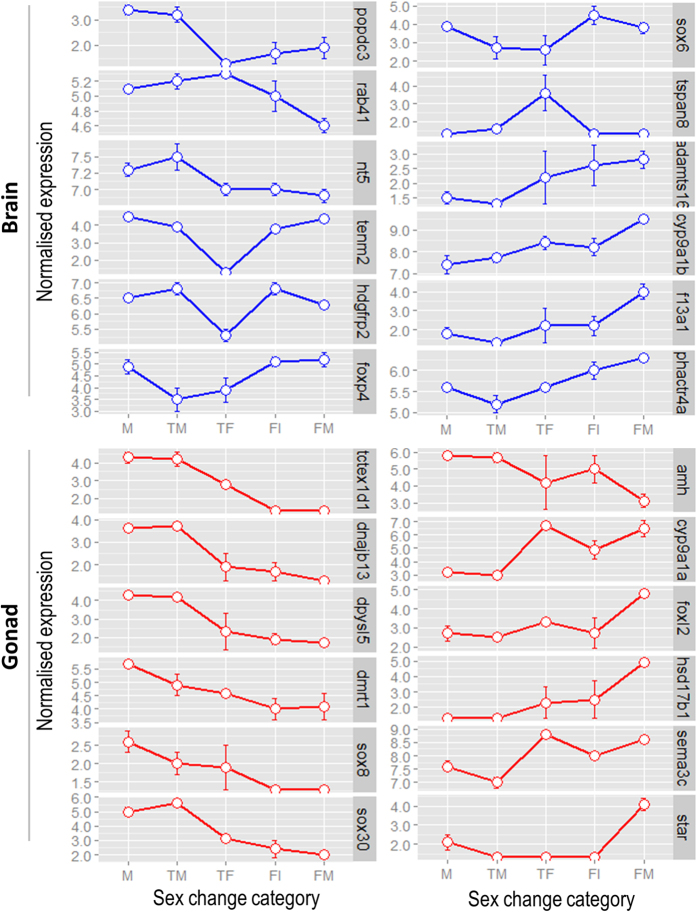
Expression of selected key genes during sex-change stages in brain and gonad in clownfish (*Amphiphrion bicinctus*). The vertical axis shows the normalized gene expression levels, bars represent the mean for each sex category ± SE. The description of each gene is found in Table 3, and the associated statistics in Supplementary Tables S2 and S3. Labels: M – males; TM – transitional males; TF – transitional females; FI– immature females; and FM – mature females.

For further analyses, we selected all contigs showing significant correlation and differential expression at p < 0.001, a total of 173 contigs from the brain and 768 contigs from the gonadal transcriptomes (Supplementary Fig. S2). The PCA and cluster analysis performed on the selected contigs from the brain revealed a clear clustering of individuals into two well-defined groups: (i) transitional fish (transitional males and transitional females) and (ii) non-transitional fish, (males, mature females and immature females) (Fig. 3). The difference between transitional males and transitional females is considerably larger than differences between any groups in the non-transitional cluster. Although males and mature females form two distinct groups, they cluster together with immature females and are clearly separated from transitional males and females. Hierarchical clustering of the transcripts resulted in three main clusters (Fig. 3B, vertical dendrogram). Two of the clusters grouped transcripts that were either expressed in all the reproductive stages tested (BG3) or were slightly down-regulated in transitional fish (BG2). The third cluster (BG1) comprised transcripts that were clearly down-regulated in the transitional stages (TM, TF). The expression of selected genes for each group is shown in Fig. 4. Expression patterns include up-regulation in males (*popdc3, rab41, nt5*), marked down-regulation in transitional stages (*hdgfrp2*, *tenm2, foxp4, sox6),* up-regulation in transitional stages (*tspan8*) and up-regulation in females (*adamts16, cyp19a1b, f13a, phactr4)*.

Clustering of the selected contigs from the gonads resulted in a completely different arrangement (Fig. 3). The PCA and cluster analysis resulted in three major clusters. Males and transitional males clustered together forming a separate group from the female stages. Within the female stages, two other distinct groups can be observed: mature females formed a separate cluster from transitional and immature ones. Hierarchical clustering of transcripts revealed five clusters (Fig. 3B, vertical dendrogram), from those up-regulated at the onset of sex change (M and TM, GG 1), to those up-regulated only in females (FM, GG5). In between, a group of transcripts that, while still up-regulated in all male categories, were also expressed in transitional females (TF, FI, GG2) and in females (GG3). Another group of transcripts were found not to be expressed in males, but showed signs of up-regulation in transitional fish and females (GG4). These clear expression patterns allow the identification of genes that likely play key roles during each stage of sex change. Expression profiles of a typical gene from each group are shown in Fig. 4, including those up-regulated in males (*tctex1*, *dnajb13*, *dpysl5*, *dmrt1*, *hsf5*) and those up-regulated in females (*foxl2*, *hsd17b1*, *star*).

### Functional Annotation and Enrichment Analysis confirms the importance of hormone levels and steroid metabolic processes during sex change in clownfish

Out of the 47,065 contigs, 81.5% had positive BLASTx similarity hits with publicly available protein sequences. The E-value distribution of the top hits revealed that 98.7% of the mapped sequences showed significant homology (less than 1.0E-10, Supplementary Fig. S3A).

For further analyses, we selected a total of 173 contigs from the brain 12 significant at p < 0.001 and 163 resulting from the pairwise differential expression analysis. Only two were shared between these two sets and 76.3% had homology previously described genes (Supplementary Table S2). From the gonadal transcriptome a total of 768 contigs were selected (Supplementary Fig. S2), 558 significantly correlated at p < 0.001 and 434 showing significant differential expression (p < 0.001) in the pairwise comparisons, with 224 contigs shared between both groups (Supplementary Fig. S2). The vast majority (92.2%) of them were successfully annotated to known protein-coding genes, hereafter referred to as genes (Supplementary Table S3).

A BLASTx top-hit species distribution indicated that 75% of the annotated contigs had the closest homology to proteins in *Stegastes partitus*, 7% to *Oreochromis niloticus*, 15% to 27 other teleost species, while the remaining 1% had close homology to proteins in other organisms, mostly marine invertebrates (Supplementary Fig. S3B).

To aid the overall functional interpretation of the response to sex change, we assigned genes to groups according to their gene ontology (GO) annotation for biological processes. At the brain level, hierarchical clustering revealed similar patterns of expression between two well-supported groups, transitional stages (transitional males and females) and non-transitional stages (males, immature and mature females) (Fig. 3). Analysis of differentially expressed contigs that were specifically up-regulated in transitional stages resulted in nine over-represented GO terms that had significant GO enrichment for regulation of the G-protein-coupled receptor protein signaling pathway and activation of meiosis involved in egg development (Supplementary Fig. S4, Supplementary Table S4).

At the gonadal level, our hierarchical clustering revealed three well-supported groups, with male stages forming a clearly separated cluster from the two female groups (Fig. 3). GO enrichment analysis was performed on differentially expressed contigs which showed negative and positive correlation to the index of sex change (for details on selected contigs, see Supplementary Table S3). Negative correlated contigs analysis resulted in 75 GO terms that were over-represented during the male stages of the sex change (Supplementary Table S5), subsequently slimmed in REVIGO to 36 GO terms. Their putative functions include reproduction and, interestingly, eye development (lens development in camera-type eye, iris morphogenesis, lens morphogenesis, embryonic retina morphogenesis in camera-type eye), suggesting the possibility of novel additional functions of these genes related to the sex change mechanism (Supplementary Fig. S5). Enrichment analysis of positively correlated contigs resulted in 58 GO-terms that were over-expressed during the female stages (Supplementary Table S6), slimmed in REVIGO to 29 GO-terms. Their putative functions include regulation of hormone levels and steroid metabolic processes (Supplementary Fig. S6).

### Validation of expression patterns by qPCR

To confirm the expression changes detected by RNA-seq, we performed qPCR on six genes exhibiting broad expression ranges: *amh*, *dmrt1, cyp19a1a* and *foxl2* in gonad and *phactr4* and *tenm2* in brain. Normalized RNA-seq expression data compared to qPCR data showed correlation coefficient values between 0.7 and 0.9. Expression patterns were consistent between both methods (Supplementary Fig. S7).

## Discussion

A few recent studies have analyzed the transcriptome of non-model fish species to shed light on sex differentiation and sex-specific gene expression. Remarkable differences in gene expression profiles of gonads and brain have been detected between males and females. Such studies include hermaphrodite species (e.g.: *Thalassoma bifasciatum*)^18^ and gonochoristic species displaying a dominance hierarchy that is similar to that of clownfish (e.g. African cichlids)^19^. However, little previous work has examined differences in gene expression across a dynamic life history process. This study addresses the transcriptome analysis of sex change in a species with a strong social hierarchy. Our study is the first to highlight the rapid and complex genomic responses of the brain and gonad associated with sex change in the natural environment. Furthermore, our work examines relative gene expression across multiple groups—rather than just two contrasting conditions—allowing us to explore the differences in expression patterns, including those of intermediate stages, during the whole process.

### The genetic mechanism underlying sex change in A. bicinctus: the central role for the aromatase gene

Our results show a transcriptional response in the male’s brain two weeks after the female’s disappearance. This response is subsequently transmitted to the gonads where differential expression and histological changes are clearly observed after three to four weeks. The main transcriptional response driving sex change at the brain level was found to be completed 30 days after removal of the original female, while differential expression is still detected in the gonads 50 days after removal of the original female, although sex change is completed when males become immature females. We hypothesize that changes in brain expression are transmitted to the gonad through the hypothalamic-pituitary-gonadal axis. Receptors on the gonadal tissue receive the hormonal signals resorbing the testicular tissue and developing the ovary to complete the gonadal sex change. Our study has revealed an important number of genes involved in these processes, some of well-known and several novel genes potentially playing a role in sex change, both at the brain and gonadal levels (summarized in Table 3, expression profiles are shown in Supplementary Fig. S8).

#### Changes at the brain level: aromatization plays an essential role during sex change

Our analysis shows that functional males (day 0) and males whose female partners were removed 5 and 11 days earlier display very similar expression profiles, while no histological signal of sex change was observed at the gonadal level. Reorganization of gonadal tissue is costly, not only energetically but in terms of the time spent on sex change, a period during which the fish are not able to reproduce. In the case of *A. bicinctus*, this reorganization is moreover irreversible due to the full degeneration of the testicular tissue. It has been shown in other *Amphiprion* species that staying male and re-pairing with a new larger mate who has emigrated from another anemone is a more affordable mate acquisition strategy^9^. Thus, the disappearance of the female would not trigger the sex change process at the brain level until it becomes clear to the male that moving up in the hierarchy is beneficial given the lasting absence of the female. Consistent with this hypothesis, we found the first clear changes in brain expression profiles of transitional males (days 15 to 30 after original female removal) compared to males (0–11 days). These individuals showed a marked down-regulation in differentially expressed genes that was maintained also in transitional females. On the other hand, females displayed very similar expression patterns to males, indicating subtle sex differences in overall gene expression between both mature stages. Accordingly, most differentially expressed genes were detected in transitional stages and only twelve showed a significant correlation to the index of sex change.

To tease out the molecular basis of sex change in clownfish at the brain level, we examined differences in neural expression of both candidate and highly differentially expressed novel genes and assessed their gene expression patterns during the whole sex change process. A very well-known gene that plays an important role in sex change in teleosts both at the gonad and brain level is *cyp19a1* (also known as aromatase or *P450aromA*). In non-mammalian vertebrates, *cyp19a1* is a key steroidogenic enzyme operating in the female pathway and converting androgens into estrogens^20^, hence controlling the balance of sex steroids. This gene followed a clear increasing trend of expression values towards the female categories, resulting in significant correlation and significant differential expression between male stages and mature females in our experiment.

Two possible candidates to be involved in the mechanism of sex change showed a significant down-regulation during transitional stages: *sox6* and *foxp4*. Sox6 is a transcription factor that has been found to be involved in the sex developmental pathway of vertebrates by regulating spermatogenesis^21^. FoxP4 is a member of the FoxP gene family of transcription regulators whose functions and targets have yet to be determined^22^. The fox genes play important roles in various biological processes, including sexual development. A transcriptomic analysis revealed very high expression levels of *foxp4* in the gonads of Nile Tilapia^23^, whereas sex-dependent changes in expression of *foxp4* affecting only male mammals were found^24^.

**Figure 5:**
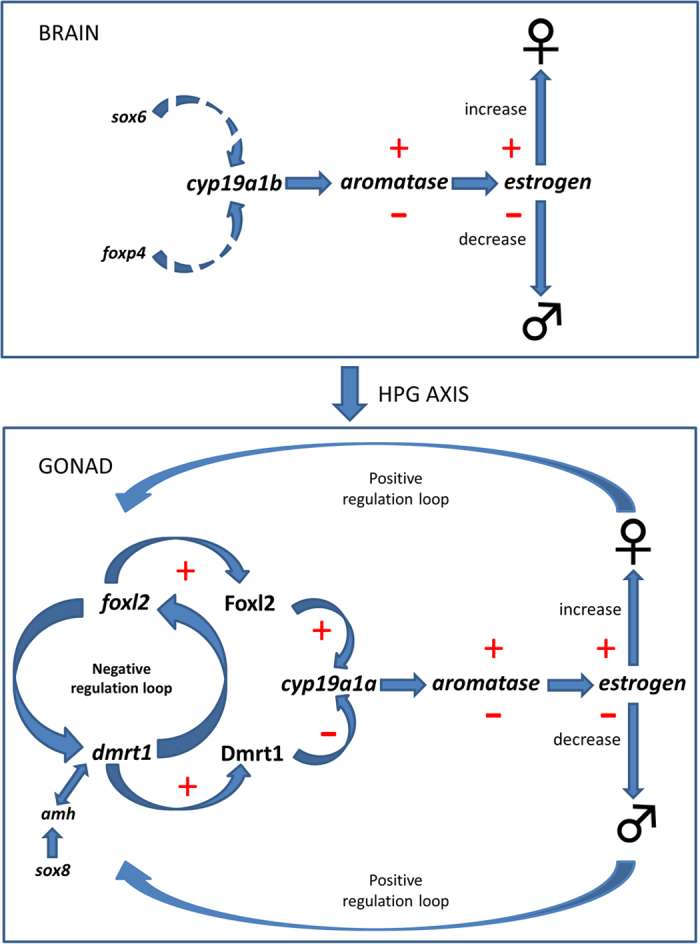
Schematic representation of the proposed genetic mechanism underlying sex change in *A. bicinctus* at the brain level (upper panel) and the gonad (lower panel). Solid arrows represent the known regulations of genes involved in sexual differentiation in *A. bicinctus*, while dashed arrows correspond to the suggested ones in females (upper part of the figure, both panels, ♀) and males (lower part of the figure, both panels, ♂). +indicates up-regulation/increase, − indicates down-regulation/decrease. Abbreviations: HPG, hypothalamic-pituitary-gonadal; cyp19a1b, cytochrome P450, family 19, subfamily A, polypeptide 1b; sox6, SRY (sex determining region Y)-box 6; foxp4, forkhead box P4; foxl2, forkhead box L2; Foxl2, forkhead box L2 protein; dmrt1/Dmrt1, doublesex and mab-3 related transcription factor 1; cyp19a1a, cytochrome P450, family 19, subfamily A, polypeptide 1a; amh, anti-Mullerian hormone; sox8, SRY (sex determining region Y)-box 8.

We propose that *cyp19a1b* plays a central role in the mechanism of sex change in the brain of *A. bicinctus* (Fig. 5) by modulating the balance between estrogen and androgen signaling. Both *sox6* and *foxp4* may play a role in regulating the expression of aromatase and/or other genes involved in the sex steroidogenic pathway in *A. bicinctus* at the brain level but the specific mechanism of action remains to be established.

Some genes encoding key neural regulators that have been shown to play major roles during sexual development and maturation in fish at the brain level such as gonadotropin-releasing hormone (GnRH), arginine-vasotocin (AVT), Kisspeptin (Kiss) or steroid hormones and their receptors, were not detected in our analysis. Note, however, that our study examined the brain as a whole, whereas it is well established that sex-specific differences in gene expression are present in fish when only certain brain regions are considered^25^. GnRH, AVT and Kiss have been shown to be involved during sex change in other *Amphiprion* species, but all the studies were restricted to the hypothalamus^26^,^27^,^28^. Similarly to our results, no significant differences in the expression levels of estrogen receptors were detected in the forebrain of bluehead wrasses^18^.

#### Changes at the gonadal level: a feedback loop between dmrt1 and foxl2 that regulates the estrogen/androgen balance drives sex change in A. bicinctus

Our analysis revealed a considerably higher number of transcripts showing both significant correlation to the index of sex change and significant differential expression in the gonads of *A. bicinctus* compared with their brains. Cluster analysis of these differentially expressed transcripts revealed three well-differentiated groups: (i) males and transitional males), (ii) transitional and immature females, and iii) mature females (Fig. 3). The main changes in expression related to sex change at the gonadal level started 24 days after the removal of the original female and were observed until the end of the experiment (day 50). Histological analysis indicated that male tissue was completely resorbed after 50 days and therefore the sex change process was completed, although none of the new females were spawning capable yet, as opposed to the original mature females.

Moreover, our study revealed a higher number of over-expressed transcripts at the gonadal level during the first stages of the sex change, when the transitional individuals were still acting as males, than at the end of the transition to becoming females. This is in accordance with the fact of *A. bicinctus* is a sequential hermaphrodite displaying ambisexual gonads with co-existing functional testicular tissues and non-functional ovarian tissues during the male-phase, whereas there are only functional ovaries in the female-phase^14^. Up-regulation or expression of a higher number of genes is required for the maintenance of the male status of the gonads (ambisexual gonad) when compared to that required for the female status (ovarian-only). This male-bias has been observed in other fish species at the gonadal level^19^,^29^,^30^.

Sexual differentiation in teleosts has been more extensively studied at the gonad than at the brain level. It is a major process that takes place after sex determination and involves the actual development of testes or ovaries from the undifferentiated gonad. Gene regulatory cascades consisting of a complex network of transcription factor interactions and signaling molecules control gonad differentiation and identity maintenance^31^. Comparative studies on vertebrates have revealed a remarkable diversity of “master sex-determining genes” at the top of the genetic cascade by which sex is determined and maintained. In contrast, downstream components operating during these processes appear to be evolutionarily more conserved^32^. This has inspired the paradigm that in sex determination and differentiation “masters change, slaves remain”^33^.

To dissect the molecular basis of sex change at the gonadal level of *A. bicinctus*, we examined differences in well-known gene candidates involved in the regulatory network of sexual development. Among the downstream genes, the *cyp19a1a* enzyme is one of the best-studied genes known to play an important role in sex change in teleosts, as mentioned earlier. In our experiment, *cyp19a1a* showed a strong up-regulation in all female categories, similar to the trend found in the brain, resulting in significant correlation and significant differential expression between male and female stages. At the gonadal level, *cyp19a1a* is considered essential for ovarian differentiation and development in fish^34^, amphibians and reptiles^35^. It has been widely characterized in gonads of sex-changing fishes, including the yellowtail clownfish^16^, and its pivotal role in gonadal sex change in teleost fish has been extensively studied^36^. The Foxl2 transcription factor also plays a decisive role in the ovarian differentiation process in various vertebrate species, including fishes. It is involved in the regulation of estrogen synthesis via direct modulation of aromatase expression and possibly the entire steroidogenic pathway^37^. This protein is also required to maintain the identity of ovarian granulosa cells^38^. In our experiment, *foxl2* was markedly up-regulated in mature females, displaying a expression profile parallel to *cyp19a1*. A positive feedback loop regulating these two genes in fish^36^ has been suggested, since *foxl2* regulates *cyp19a1*, while estrogens up-regulate *foxl2*^37^. Besides the *cyp19a1a* gene, another target of *foxl2* in the ovaries is the steroidogenic acute regulatory protein (Star) whose transcriptional activity is repressed by Foxl2 in mice^39^. In our experiment, a transcript encoding Star was markedly up-regulated in mature females, resulting in differential expression compared to all the other categories. This protein controls the rate-limiting step in steroid hormone synthesis and is required for normal ovarian steroid production in mice and humans^40^. Similarly, *hsd17b1* was also found to be significantly up-regulated in mature females, showing significant differential expression compared to all other categories and significant correlation to the index of sex change. This gene encodes the enzyme estradiol 17β-dehydrogenase 1, converting low-activity estrone to high-activity 17β-estradiol in fish and in mammalian ovarian granulosa cells^41^ and, along with *cyp19a1*, catalyzes the final steps in estradiol biosynthesis from theca cell-derived androgens^42^. All these genes are key players that regulate ovarian steroidogenesis.

On the other hand, our analysis revealed three genes known to have a clear role in sex determination, testicular differentiation and spermatogenesis in teleosts: *sox8*, *dmrt1* and *amh*^43^,^44^. These three genes were strongly up-regulated in males compared to females in our study, showing a strong negative correlation to the index of sex change. Recent work has revealed that Sox8 is an important determinant for the maintenance of testis cell identity in mice^45^ as well as a critical regulator of adult Sertoli cell function and male fertility^46^.

Sox8 regulates the expression of *amh* in mice^47^, which encodes a member of the transforming growth-factor-beta gene family that mediates male sexual differentiation and development. In our study, two different transcripts encoding Amh were detected (*amh1* and a*mh2*), both displaying a steady decrease in expression values throughout the sex change. It has been proposed that Amh is an important hormone involved in the sex change of the protandrous black porgy^48^. In zebrafish, estrogen-induced alterations had a suppressive effect on the expression of *amh* and *dmrt1* and this was found to be associated with a disruption in male gonadal sex development^49^.

On the other hand, *dmrt1* had a marked correlation with the index of sex change with a steady decrease in expression values. The *dmrt1* gene family has been found to be involved in sexual differentiation in organisms as phylogenetically divergent as corals, *Caenorhabditis elegans*, *Drosophila*, fishes, frogs, birds and mammals^50^. In fishes, either male-restricted or strong male-biased expression has been reported in at least twenty gonochoristic species (see review^50^ and references therein). In sequential hermaphrodites, its expression has been shown to parallel the development and regression of the testis, both in protandrous^51^ and protogynous species^52^. A recent study has highlighted the important role of Dmrt1 and Foxl2 in maintaining sexual cell identity. The loss of Dmrt1 in male adult mice can cause sexual transdifferentiation of testicular Sertoli cells to ovarian granulosa cells. Similarly, *dmrt1* expression in the ovary of mice, even in the absence of the testis-determining gene *sox8*, silenced *foxl2* and reprogrammed adult granulosa cells into Sertoli-like cells^53^.

Based on our results, we postulate that *cyp19a1* and *foxl2* play a pivotal role in the activation of the female pathway driving the gonadal transformation from testis to ovary during sex change in *A. bicinctus*, while Sox8, Dmrt1 and Amh are important for testis maintenance. All these genes have shown consistent expression profiles in previous studies of other sex-changing species, including protandrous (sharpsnout seabream^29^, Asian seabass^54^, black porgy^55^) and protogynous fish (bluehead wrasse^18^). Similarly to our study, comparison of female and male gonads in all four species have revealed a male-biased expression for *dmrt1, amh* and *sox8*/*sox9*, while *cyp19a1* and *foxl2* showed a female-biased profile in all but Asian seabass.

We propose a feedback loop between *dmrt1* and *foxl2* that regulates the production of the *cyp19a1a* enzyme and, by implication, the estrogen/androgen balance, hence controlling gonad identity change in *A. bicinctus.* The proposed mechanism involves a complex regulatory loop combining transcriptional regulation with steroid hormonal activity (Fig. 5).

Similarly to the brain, some well-known gene candidates involved in the regulatory network of sexual development were absent from our analysis. Wnt signaling has been implicated in a reproductive role in teleost, promoting ovarian differentiation through the upregulation of gonadal aromatase^56^. Our data did not reveal significant differences in expression throughout sex change of key members of the Wnt-signaling cascade, such as wnt2, wnt4, ctnnb1 (beta-catenin 1), or rspo-1. However, a female-specific function of the Wnt pathway does not seem to be a general pattern in fishes^57^. In addition, some key genes implicated with testicular differentiation in other fish species like wt1, gsdf or nr5a2 showed no sex-biased expression in our study. These genes are likely to represent early testis genes^1^,^58^,^59^ and might therefore not be involved in sex change in this protandrous species. However, we cannot exclude the possibility of having missed these genes in the annotation process due to a mutation, sequencing error or simply due to the limited genomic information available in the public databases for the non-model organism under study.

### Novel genes in the context of sex change at the brain level

Several genes with a potential role in the sex change process were found to be down-regulated during the transitional stages, among them the gene encoding teneurin-2 (*tenm2*). Teneurins are a unique family of transmembrane proteins conserved from *C. elegans* and *D. melanogaster* to mammals, which are predominantly expressed in the central nervous system^60^. Interestingly, a recent study proposed that Tenm2 may have an effect on the sexual maturity of chickens since the expression of genes in the nervous system can influence the age when chickens lay their first egg^61^. The up-regulation of teneurin-2 expression observed in our experiment in mature stages (both mature males and females) may indicate that it plays a role in the mature gonads in *A. bicinctus*.

The transcript encoding the hepatoma-derived growth-factor-related protein 2 (*hdgfrp2*) was also down-regulated during transitional stages, resulting in significant differential expression between transitional females and all the other categories. This gene is expressed in a wide range of tissues suggesting a function in cells of the central nervous system, but very little is known about the function of Hdgfrp2^62^. Interestingly, over-expression of *hdgfrp2* was detected in male brain tissue of *E. cyanostictus* in a study comparing the transcriptomes of male and female East African cichlids^19^.

The transcript encoding coagulation factor XIII A chain-like (*f13a*) showed the highest positive correlation to the index of sex change in our study and strong up-regulation in mature females, resulting in significant differential expression compared to all the other categories. Female-biased expression of *f13* was also detected in a transcriptomic analysis of the guppy brain^63^. F13 is best known for its role in fibrin stabilization during coagulation, but recent advances have proposed a novel function in neuronal regeneration in fish^64^. Similarly, the transcript encoding the disintegrin and metalloproteinase with thrombospondin motifs 16 (*adamts16*) showed a strong correlation to the index of sex change. Adamts16 is a member of a family of metalloproteinases, expressed at high levels in adult brain and ovary in humans^65^. A recent study has revealed that it is essential for normal development of the testis in rats^66^.

On the other hand, the transcripts encoding 5′-nucleotidase (*nt5*), popeye domain-containing protein 3 (*popdc3*) and *rab41*, were the only genes in the present study that showed a significant negative correlation to the index of sex change. Nt5 represents the major enzyme responsible for the formation of extracellular adenosine, involved in the regulation of neurotransmitter release^67^. Gender-dependent changes have been shown for *nt5* activity and nucleoside transport in rodents^68^ while gender differences in nucleoside levels in the brain have been detected in humans^69^.

Popdc3 belongs to a family that codes for membrane proteins that are expressed in neuronal cells^70^ of unknown function, while Ras proteins are involved in signal transduction pathways of several cellular functions, as cell proliferation, differentiation and apoptosis^71^.

The expression of tetraspanin 8 (*tspan8*) was significantly up regulated in transitional females in our study. Tspan-8 is known to mediate signal transduction events that contribute to the regulation of cell development, activation, growth and motility^72^. Interestingly, genetic ablation of *tspan8* in mice resulted in a reduction in the body weight of males fed a normal diet, suggesting that it may have a role in the neural control of metabolism^73^. This last function might explain the up-regulation observed in the transitional stages in our study, since sex change implies the rapid growth of the sex-changing individual who is freed from the strict social constraints previously imposed by the dominant female pair mate.

Finally, the transcript encoding phosphatase and actin regulator 4A (*phactr4a*) showed significant differential expression between male and female stages and positive correlation to the index of sex change in our experiment. Phactr4a is critical during neurulation and eye development^74^. Its role in the brain of teleosts remains unexplored.

### Candidate and novel genes in the context of sex change at the gonadal level

Several other known candidate genes that may play a role in gonadal development were detected in our study. The transcript level of several Ras factors (*rassf1*, *rassf8*, *arhgef25*, *arhgap29*) was found to be significantly up-regulated during the female stages (immature and/or mature females) in our study. These genes participate as central control elements in signal transduction pathways and their functions are related to cell growth/arrest, differentiation and apoptosis. A recent study has demonstrated direct regulation of Ras proteins by progesterone receptor^75^ while their activation in granulosa cells has been shown to be crucial for normal follicle development^76^.

Similarly, several genes with immune-related function were up-regulated in *A. bicinctus* females. It has been proposed that immune molecules may play an essential role in the orchestration of gametogenesis and the maintenance of gonad tissue homeostasis in fish^77^. The *lrig1* (leucine-rich repeats and immunoglobulin-like domains protein 1) transcript and other differentially expressed genes related to the immune system (*ildr2*, *c1qtnf4*) were highly up-regulated in mature females. Studies of several fish species have shown that maternal immunoglobulins (*ig*) are transferred from mother to progeny in order to confer higher survival to the larval offspring^78^. Up-regulation of immune-related genes observed in *A. bicinctus* females in our study could be similarly related to the transfer of immune components to the progeny and/or the mother’s own immune system.

Several genes encoding collagen-alpha-chains (*col4a4, col4a5, col15a1*) showed a strong positive correlation to the index of sex change in our study. These genes have a function in the development of ovarian follicles, both in fish and mammals^79^.

Other genes showing a strong female-biased expression were *fstl1* (follistatin-like 1) and semaphorin-3C (*sema3c*). Similarly to what has been found in rainbow trout^80^, the spatio-temporal expression pattern of *fstl1* in clownfish is intimately coupled to *cyp19a1* expression. Sema3c is expressed in granulosa cells and required for the process of follicle expansion in mammals^81^. Both proteins may play a significant role in ovarian function by modifying steroidogenesis in the follicles.

On the other hand, the expression of a number of genes was found to be male-biased, including the *t*-*complex* testis-expressed 1 (*tctex1*), highly up-regulated in the male stages in *A. bicinctus*, resulting in significant differential expression between male categories compared to all female stages and one of the strongest negative correlations observed in our study. This gene, encoded within the *t-complex*, a chromosomal region containing genes known to specifically influence male fertility^82^, is an important molecular component of spermatogenesis. Similar to our findings, *tctex1* was found to be more up-regulated in testis of *T. thynnus* compared to in ovarian tissues^83^.

Sox30 is a member of Sry-related HMG box (Sox) transcription factors, involved in spermatogonial differentiation and spermatogenesis^84^. In our study, the transcript encoding Sox30 was significantly up-regulated in male categories compared to females and showed a strong negative correlation to the index of sex change.

The transcript encoding the dnaJ homolog subfamily B member 13 (*dnajb13*) showed a strong negative correlation to the index of sex change and significant differential expression between male categories and all the female stages. The product of this gene participates in spermiogenesis and ensures the motility of mature spermatozoa in mice^85^. Dnajb13 belongs to the heat-shock protein 40 family and the same expression pattern was found for other transcripts encoding heat shock factors (Hsf5, Hsf2bp). This family includes proteins that are closely related with gonad development and regulation of spermatogenesis and they may also play a role in suppressing the occurrence of apoptosis in testis^86^. The decreasing expression values throughout the sex change process observed in our study would therefore favor apoptosis and testis resorption.

Interestingly, a number of genes (e.g., *syne2* and *dpysl5*) with functions related to eye development were detected as differentially expressed in our study. Nesprin-2 (Syne2) belongs to a family of proteins with multiple functions, including a role in maintaining the organization and structural integrity of the nucleus^87^. This gene is also required for nuclear migration during the development of the retina^88^. Although numerous isoforms of *Syne2* have been detected in several tissues in humans, including testis^89^, their specific functions are not known. In our study, *syne2* showed a strong up-regulation in immature and mature females, resulting in significant differential expression between male categories and mature females and significant correlation to the index of sex change. Dpysl5 plays a role in neuronal differentiation, axon growth and synaptic plasticity^90^ and it has also been identified in the retina and optic nerve^91^. High expression levels have been detected in ovarian follicles and testis in humans, but its specific role in the gonads, if there is one as suggested by this study, is unknown. In our experiment, *dpysl5* was significantly up-regulated across the male stages compared to all female stages and showed a strong negative correlation with the index of sex change. Moreover, the GO analysis showed enrichment in several categories related to eye development (lens morphogenesis, lens and lens induction in camera-type eyes, iris morphogenesis) reinforcing the possibility of a previously undescribed role of these genes during gonadal sex change in *A. bicinctus*.

The transcriptome dataset generated here represents a valuable genomic resource for screening potential gene targets involved in the sex change process. Our analysis has provided a good picture of the main mechanism underlying sex change in *A. bicinctus*, as well as detailed information on specific genes involved during every step of the process. It has unveiled both well-known and as yet *A. bicinctus* uncharacterized genes associated with sex change as well as potential regulatory target genes.

## Methods

### Site, Sample Collection and Experimental Design

This SCUBA dive-based study was carried out between November 2012 and July 2013. Sixteen clownfish families were localized and tagged *in situ* on the exposed side of Al-Fahal reef, located 13 km off the Saudi Arabian coast in the Central Red Sea (N22.18.333, E38.57.768; see Supplementary Fig. S9).

All selected families were living at depths from 4 to 20 meters and consisted of at least three individuals (the breeding pair and from 1 to 4 additional immature individuals). All individuals were measured (total length, TL, to nearest 0.1 mm) using calipers underwater and the families were selected based on a minimum size for the breeding male of 50 mm (see Supplementary Table S7). Fishes were individually tagged by injecting fluorescent visible implant elastomer (VIE, Northwest Marine Technology Inc, Tumwater, WA) under the surface of their skin. These tags have no adverse effects on individual growth or survival. Additionally, numbered buoys were used to mark each family and corresponding host anemone. To reduce the stress related to the tagging process, families were not approached for two months after the last family was tagged.

**Figure 6:**
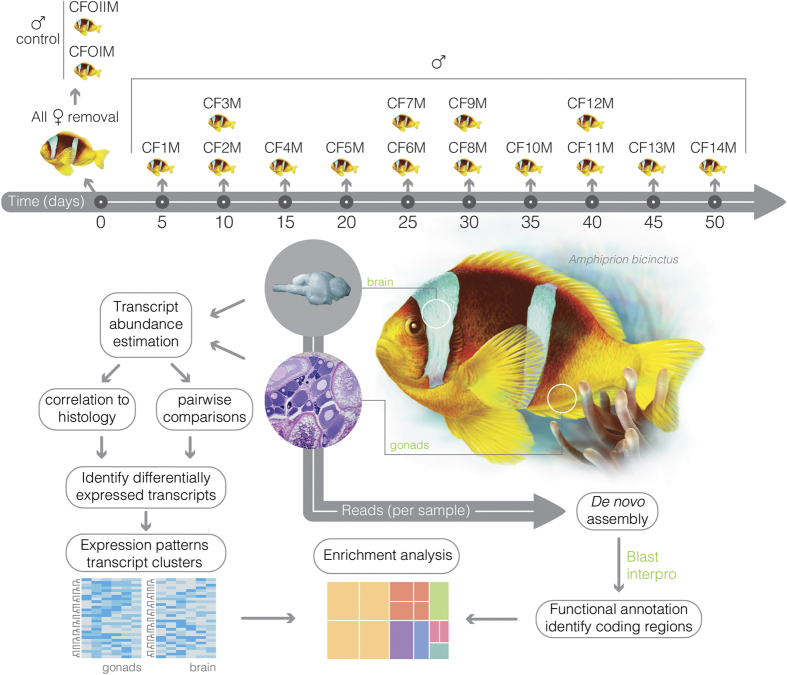
Diagram showing the experimental design used to profile molecular events related to sex-change in *A. bicinctus*. Top: sixteen clownfish families were involved in the study, two functional males and all females were removed at time point 0 to trigger the sex-change; individual codes and the planned time point for collection (days after female removal) of the 14 sex-changing individuals along the 50-day experimental period is illustrated; bottom: flowchart depicting the transcriptome analysis pipeline.

Sex change was triggered by the removal of the dominant female, i.e., the largest individual from all families. Transcriptional responses were then assessed by collecting one sex-changing individual approximately every 5 days for 1.5 months to cover the full time course of the sex change process (Fig. 6). Extra fish were collected when possible in order to prevent potential problems during sample treatment (RNA isolation, sequencing, histology) and ensure the full coverage of the sex change process. The date of sacrifice, length, and time lapse since female removal are enumerated in Supplementary Table S7.

In all, two functional males, 14 sex-changing individuals and corresponding 16 female partners were collected on SCUBA with hand nets. They were sedated with a clove-oil mixture (10% clove oil (Sigma-Aldrich, St. Louis, MO), 5% ethanol, 85% sea water), carried to the boat in plastic zip-lock bags wrapped in black plastic to avoid stress from sunlight, and immediately processed on board. Fishes were euthanized using clove oil and the brain and gonads were surgically removed. The brain and one gonadal lobe were preserved in RNA*later* (Ambion, Austin, TX) for transcriptomic analysis. Both organs were processed on the same day upon arrival at the laboratory. The second gonadal lobe was fixed and preserved in 10% buffered formalin (Sigma-Aldrich, St. Louis, MO). Fish bodies were stored on dry ice during transportation and immediately stored at −80 °C upon arrival at the laboratory.

This research was carried out under the general auspices of King Abdullah University of Science and Technology’s (KAUST) arrangements for marine research with the Saudi Arabian Coast Guard and the Presidency of Meteorology and Environment. The animal use protocol was approved by KAUST’s Biosafety and Ethics Committee (KAUST does not provide specific approval number). The methods in this research were carried out in accordance with the approved guidelines.

### Histological Analysis of gonadal developmental stages

Fixed gonad tissues were dehydrated, embedded in paraffin, sectioned at 3 μm, and stained following the standard protocol of haematoxylin-eosin for histological examination^92^. Serial cross sections were performed in two areas of the gonad, the central and posterior regions, to account for differences in tissue distribution along the gonad. A high-resolution image of at least one section in each area was taken so that the whole cross section was visible in the image.

Functional males were identified by the presence of well-developed testicular tissue containing seminiferous tubules with cells in all stages of development and ovarian tissue containing only primary-growth oocytes (Supplementary Fig. S10A). Females were identified by the absence of testicular tissue and the presence of secondary-growth oocytes or spawning indicators (Supplementary Fig. S10D).

Sex change was assessed by estimating the proportion of testicular tissue in each section, as each degenerating testis steadily shrinks throughout the sex change process (Supplementary Fig. S10B,C). Image analysis was used to measure the whole gonadal area and the area occupied by testicular tissue in each specimen (males, transitional and inmature individuals). The proportion of gonad occupied by testicular tissue was used as an index of sex change since testicular tissue is absent in mature females or it is minimal.

### Total RNA extraction and cDNA library construction

Fish brains and the gonad preserved in RNA*later* were weighed and placed in RNA-free microcentrifuge tubes containing 1 ml of TRIzol Reagent (Invitrogen, Carlsbad, CA) for RNA isolation. When these tissues weighed more than 10 mg, they were split and pooled together at the end of the procedure. The tissues were homogenized using a cordless motor pestle (Thermo Fisher Scientific, Waltham, MA) until no pieces were visible. The lysates were incubated for 5 min at room temperature to allow complete dissociation of nucleoprotein complexes. Phase separation was accomplished by using 0.2 ml of chloroform (Thermo Fisher Scientific, Waltham, MA) per tube and centrifuged at 12000 g for 15 minutes at 4 °C. An equal volume of 100% ethanol was added before proceeding to binding, washing and eluting using the PureLink RNA Mini Kit (Life Technologies, Carlsbad, CA) following the manufacturer’s instructions. Total RNA was eluted in 50 μl of RNase-free water (Ambion, Austin, TX).

Quality and concentration of total RNA were checked using a Bioanalyzer 2100 (Agilent, Santa Clara, CA) and a Qubit fluorometer (Invitrogen, Carlsbad, CA) prior to library creation. Poly-A mRNA was purified from 3 μg of total RNA using a Dynabeads Oligo (dT) Kit (Life Technologies, Carlsbad, CA) and following a scaled-down version of the manufacturer’s protocol. To assemble the next-generation sequencing (NGS)-based RNAseq, we generated 2×100 bp sequence libraries using the NEBNext Ultra Library Prep Kit for Illumina sequencing platforms (Illumina, San Diego, CA) according to the manufacturer’s instructions and using 12 cycles of PCR for enrichment. The libraries were checked for quality on a Bioanalyzer 2100 DNA 1000 chip and quantified using a KAPA Library Quantification Kit for Illumina sequencing platforms (Kapa Biosystems, Wilmington, MA). Quantitative PCR (qPCR) was performed as per the manufacturer’s protocol. Based on these results, mRNA sequencing libraries were multiplexed in equal quantities (10 nM) and sequenced on four lanes on a HiSeq 2000 platform (Illumina, San Diego, CA) at the KAUST Bioscience Core Laboratory (Thuwal, Saudi Arabia). In total, 40 samples were sequenced corresponding to the brain and gonad of the two functional males, twelve of the fourteen sex-changing individuals (two were excluded due to low RNA quality, see Supplementary Table S7 for details) and six of the females.

### Read pre-processing, de novo transcriptome assembly and abundance estimation

The flowchart depicting the transcriptome analysis pipeline is shown in Fig. 6. The quality of RNA-seq reads was checked and visualized with FastQC. Each raw read was trimmed at the first base with a mean quality score threshold (Q-score) of less than 20 and the 3′ end was discarded using custom Java scripts. The pre-processing also included the removal of the sequence adaptors. Fragments with both surviving read pairs were then subjected to *in silico* normalization using Trinity^93^ (version: r20140717).

Two *de novo* assemblies were generated using ABySS v1.3^94^ and Trinity. Based on the number and length of the produced contigs, Trinity was selected for subsequent analyses.

The assembled transcriptome was evaluated for completeness, using the widely conserved set of 248 core eukaryotic genes dataset (CEGs)^95^. Sequence comparisons were conducted using NCBI’s BLASTx and bit-scores ≥50 were considered significant.

The reconstructed contigs were further filtered for the expression analysis using TransDecoder^93^, which identifies candidate-coding regions. Similar contigs with sequence identity thresholds of 90 were merged using CD-HIT-EST 4.5.3^96^ to condense the number of contigs.

The paired reads of each sample were mapped to the assembly using BWA v0.7.5^95^. We then used SAMtools^96^ v1.1 to quantify mapped read counts for each contig per sample. Raw read counts for each contig were normalized using DESeq^97^ (version 1.18.0) and extracted for analysis at the gene level. A cluster analysis was performed to investigate the relationship between the samples. Sample-to-sample distances were estimated by Bray–Curtis dissimilarity (R^98^, *vegan* 2.2-1 package) followed by hierarchical clustering performed in R (*stats* 3.1.3 package). Heatmaps were generated using heatmap.2 (R package *gplots*, version 2.160).

### Differential expression analysis

Both types of tissue (brain and gonad) grouped into two well-differentiated clusters and were therefore analyzed separately. Two different approaches were taken to analyse the differences in expression among individuals during sex change:

- To detect trends in gene expression tightly correlated with the gonadal developmental stage throughout the sex change process (i.e., genes whose expression values follow a linear increasing or decreasing trend during the time course of sex change), the Pearson correlation coefficients between normalized expression levels (DESeq_normalized) and the index of sex change of each individual (i.e., the percentage of female tissue in the gonad; see Table 1 in Results) were calculated for all 47,065 contigs in the final dataset. The most significantly correlated contigs were selected for further analysis, resulting in a list of contigs positively and negatively correlated with the histology data. Correlations were estimated using the *psych* package in R and p-values were adjusted using Benjamini & Hochberg adjustment method.
- To detect differentially expressed genes among the different stages of the sex change process, pairwise comparison was performed, since the correlation analysis is unable to detect differences in expression in transitional fish because correlation requires linearity. For this analysis, samples were grouped in different stages, according to the percentage of ovarian tissue in the gonads. Differentially expressed contigs were identified using DESeq. Multiple test correction was performed using *p.adjust* (Benjamini & Hochberg method) in R.

The correlation approach allows detecting gradual and steadily changes in expression (up- and down-regulation), which are not easily detected by pairwise analysis; while the pairwise tests identifies differences that do not follow a linear increasing or decreasing trend during the time course of sex change.

### PCA and cluster analyses of differential expression

To examine how transcriptomes from different stages were related, Principal Component Analysis (PCA) followed by hierarchical clustering was performed in R (*stats* package) on each tissue with the selected contigs (significantly correlated and differentially expressed). Heatmaps were generated using heatmap.2 (R package *gplots*).

### Functional Annotation and Enrichment Analysis

To annotate the assembled contigs, we conducted a BLASTx similarity search against a locally installed NCBI protein database, *nr* (release date 15 Oct 2014), using a bit-score of 50 as the minimum threshold.

InterProScan^99^ v5.7–48 was used to detect potential encoded proteins insufficiently represented in the databases and therefore difficult to detect by homologies to known proteins. Protein domains were searched in the Pfam^100^ v27.0 and SUPERFAMILY^101^ v1.75 databases.

An enrichment-GO analysis was performed on the subsets of genes showing differential expression between sex-changing stages to explore their functions. Over-represented domains and gene ontologies were checked using the cumulative hypergeometric test for the described subsets of contigs and p-values were adjusted using the Benjamini and Hochberg False Discovery Rate (FDR) using the Biological Networks Gene Ontology tool (BINGO, plugin in *Cytoscape*^102^ *3.2.1*) and slimmed with REVIGO^103^ software.

### Validation of expression patterns by RT-qPCR

Six genes showing differential expression in the transcriptome analysis were validated through quantification by qPCR. The primers (Supplementary Table S8) were designed according to Illumina sequencing data with Primer3^104^ (version 0.4.0). The total RNA used in qPCR analysis was the same as that used in the Illumina sequencing assay. The assay was run on the ABI 7900HT Fast Real-Time PCR system (ABI, USA) using the SuperScript III Platinum SYBR Green One-Step qRT-PCR Kit (Invitrogen, Waltham, MA), following a scaled-down version of the manufacturer’s protocol. Beta-actin was used as an internal control. The stability of its expression was previously established by analyzing 10-fold dilution series of brain and gonadal samples from individuals representing the whole range of the sex change index. All samples were analyzed in triplicate and the expression of target genes was calculated as relative fold values using the comparative *C*_T_ method.

### Availability of supporting data

This Transcriptome Shotgun Assembly project was deposited at DDBJ/EMBL/GenBank under accession number GDVCV00000000. The version described in this paper is the first version, GDVCV01000000. Raw sequence reads can be found in the SRA database under BioProject PRJNA261388.

## Additional Information

**How to cite this article**: Casas, L. *et al.* Sex Change in Clownfish: Molecular Insights from Transcriptome Analysis. *Sci. Rep.* **6**, 35461; doi: 10.1038/srep35461 (2016).

## Supplementary Material

Supplementary Information

Supplementary Tables

## Figures and Tables

**Table 1:** Histological gonad assessment of male-to-female sex change of clownfish (*Amphiphrion bicinctus*).

Individual code	Time lapse	% Male tissue	% Female tissue	Sex-change category	Individual label
CF1M	5	46.60	53.40	Male (M)	M1
CF10M	35	46.42	53.58	Male (M)	M2
CF2M	11	45.96	54.04	Male (M)	M3
CF0IM	0	45.36	54.64	Male (M)	M4
CF0IIM	0	44.33	55.67	Male (M)	M5
CF5M	21	40.03	59.97	Transitional male (TM)	TM1
CF6M	26	39.68	60.32	Transitional male (TM)	TM2
CF8M	29	35.60	64.40	Transitional male (TM)	TM3
CF4M	15	35.34	64.66	Transitional male (TM)	TM4
CF9M	31	23.54	76.46	Transitional female (TF)	TF1
CF7M	24	17.87	82.13	Transitional female (TF)	TF2
CF14M	50	12.62	87.38	Immature Female (FI)	FI1
CF11M	41	12.08	87.92	Immature Female (FI)	FI2
CF13M	47	9.01	90.99	Immature Female (FI)	FI3
CF1F	—	0	100	Mature female (FM)	FM1
CF12F	—	0	100	Mature female (FM)	FM2
CF3F	—	0	100	Mature female (FM)	FM3
CF5F	—	0	100	Mature female (FM)	FM4
CF9F	—	0	100	Mature female (FM)	FM5
CF0IF	—	0	100	Mature female (FM)	FM6

Individual code, time lapse (days between the removal of the female and the collection of the partner undergoing sex change), percentage of male and female tissue obtained by histological analysis of the individual gonads, assigned sex-change category, corresponding individual label.

**Table 2:** Number of contigs showing significant differential expression (DECs, p < 0.001) in gonad and brain throughout the sex change process in clownfish (*Amphiphrion bicinctus*).

Pairwise comparison	Number of DECs	
	Brain	Gonad
M-TM*	20	6
M-TF	14	38
M-FI	5	54
M-FM	5	167
TM-TF	23	31
TM-FI	13	34
TM-FM	66	208
TF-FI	12	20
TF-FM	23	49
FI-FM	9	46

Five categories, based on the percentage of female tissue in the gonad, were used in the comparisons, with labels corresponding to: M- male; TM-transitional males; TF- transitional females; IF- immature females and MF- mature females.

^*^See Table 1 for sex change category labels.

**Table 3:** Candidate and novel genes potentially playing a role in sex change in *A. bicinctus.*

Gene symbol	Gene name	Brief description of function	Location
*adamts16*	ADAM metallopeptidase with thrombospondin type 1 motif, 16	Essential for normal development of the testis in rats; involved in zebrafish embryos somitogenesis.	Brain
*cyp19a1b*	cytochrome P450, family 19, subfamily A, polypeptide 1a	Key steroidogenic enzyme that converts androgens into estrogens, plays an essential role in the development and sexual differentiation of the brain in vertebrates.	Brain
*f13a*	coagulation factor XIII, A	Multifunctional protein involved in regulatory mechanisms and construction and repair processes, role in neuronal regeneration	Brain
*foxp4*	forkhead box P4	Involved in brain development. Fox genes play important roles in various biological processes, including sexual development.	Brain
*hdgfrp2*	hepatoma-derived growth factor-related protein 2	Function in cells of the central nervous system that might include proliferation as well as cell survival	Brain
*nt5*	5′-nucleotidase	Enzyme responsible for the formation of extracellular adenosine, involved in the regulation of neurotransmitter release.	Brain
*phactr4a*	phosphatase and actin regulator 4a	Plays a key role in regulating synaptic activity and dendritic morphology in the nervous system, regulates integrin signaling and cytoskeletal dynamics and is critical during neurulation and eye development.	Brain
*popdc3*	popeye domain containing 3	Membrane protein that is expressed in neuronal cells in restricted areas of the brain, its function remains completely unstudied.	Brain
*rab41*	RAB41, member RAS oncogene family	Involved in signal transduction pathways of several normal cellular functions, as cell proliferation, differentiation, adhesion, apoptosis, and migration.	Brain
*sox6*	SRY (sex determining region Y)-box 6	Involved in the sex developmental pathway of vertebrates regulating spermatogenesis, modulates brain development and behavior.	Brain
*tenm2*	teneurin transmembrane protein 2	Involved in neuronal pathfinding and synaptogenesis. It might also have a role in the specification of neuronal circuits in the visual and olfactory systems	Brain
*tspan8*	tetraspanin 8	In the brain, involved in neurite outgrowth, synapse formation and in the regulation of synaptic plasticity. Tspan-8 mediates signal transduction events that contribute to the regulation of cell development, activation, growth and motility.	Brain
*Amh*	anti-Mullerian hormone	Mediates male sexual differentiation and development.	Gonad
*arhgap29*	Rho GTPase activating protein 29	Rho/Rac proteins are involved in a wide variety of cellular functions such as cell polarity, vesicular trafficking, the cell cycle and transcriptomal dynamics.	Gonad
*arhgef25*	Rho guanine nucleotide exchange factor (GEF) 25	Plays an important role in cytoskeleton organization and is therefore involved in cell adhesion, migration, proliferation, survival and differentiation.	Gonad
*c1qtnf4*	C1q and tumor necrosis factor related protein 4	Acts as inflammatory regulator and as a central regulator of food intake and energy balance.	Gonad
*col4a4*	collagen, type IV, alpha 4	Function in the development of ovarian follicules, both in fish and mammals.	Gonad
*col4a5*	collagen, type IV, alpha 5		
*col15a1*	collagen, type XV, alpha 1		
*cyp19a1a*	cytochrome P450, family 19, subfamily A, polypeptide 1b	Key steroidogenic enzyme that converts androgens into estrogens in vertebrates, essential for ovarian differentiation and development in fish.	Gonad
*dmrt1*	doublesex and mab-3 related transcription factor 1	Involved in male gonadogenesis and differentiation.	Gonad
*dnajb13*	DnaJ (Hsp40) homolog, subfamily B, member 13	Participates in spermiogenesis and ensures the motility of mature spermatozoa.	Gonad
*dpysl5*	dihydropyrimidinase-like 5	Function in neuronal differentiation, axon growth and synaptic plasticity and it has also been identified in retina and optic nerve.	Gonad
*foxl2*	forkhead box L2	Regulates estrogen synthesis via direct modulation of aromatase expression and possibly the entire steroidogenic pathway, required in order to maintain the identity of ovarian granulosa cells.	Gonad
*fstl1*	follistatin-like 1	Involved in attenuation of oxidative stress and inflammatory response.	Gonad
*hsd17b1*	hydroxysteroid (17-beta) dehydrogenase 1	Converts low-activity estrone to high-activity 17β-estradiol in fish and in mammalian ovarian granulosa cells and thus, along with cyp19a1 catalyzes the final steps in estradiol biosynthesis from theca cell-derived androgens.	Gonad
*hsf2bp*	heat shock transcription factor 2 binding protein	Related with gonad development and regulation of spermatogenesis and may also play a role suppressing the occurrence of apoptosis in testis.	Gonad
*hsf5*	heat shock transcription factor family member 5	Related with gonad development and regulation of spermatogenesis and may also play a role suppressing the occurrence of apoptosis in testis.	Gonad
*ildr2*	immunoglobulin-like domain containing receptor 2	Plays a role in the immune response.	Gonad
*lrig1*	leucine-rich repeats and immunoglobulin-like domains protein 1	Involved in signal transduction, cell proliferation, cell apoptosis, cell cycle, cell migration, and cell invasion.	Gonad
*rassf1*	Ras association (RalGDS/AF-6) domain family 1	Central control elements in signal transduction pathways with functions related with cell growth/arrest, differentiation and apoptosis. Progesterone receptor directly regulates Ras proteins and they are crucial for normal follicle development.	Gonad
*rassf8*	Ras association (RalGDS/AF-6) domain family 8	Positive regulator of cell death.	Gonad
*sema3c*	sema domain, immunoglobulin domain (Ig), short basic domain, secreted, (semaphorin) 3C	Required for the process of follicle expansion in mammals.	Gonad
*sox8*	SRY (sex determining region Y)-box 8	Is an important determinant for the maintenance of testis cell identity in mice as well as a critical regulator of adult Sertoli cell function and male fertility.	Gonad
*sox30*	SRY (sex determining region Y)-box 30	Involved in spermatogonial differentiation and spermatogenesis.	Gonad
*Star*	steroidogenic acute regulatory protein	Controls the rate-limiting step in steroid hormone synthesis and is required for normal ovarian steroid production.	Gonad
*syne2*	spectrin repeat containing, nuclear envelope 2	Role in maintaining the organization and structural integrity of the nucleus. Also required for nuclear migration during the development of the retina.	Gonad
*tctex1*	Tctex1 domain containing 1	Important molecular component of spermatogenesis, known to specifically influence male fertility.	Gonad
