# Supplementary material for: Sex Change in Clownfish: Molecular Insights from Transcriptome Analysis
Source: Sci Rep. 2016 Oct 17;6:35461. doi: 10.1038/srep35461 (PMC5066260; doi:10.1038/srep35461)
Supplement: Supplementary Information [file srep35461-s1.pdf]

Sex Change in Clownfish: Molecular Insights from Transcriptome Analysis

Laura Casas, Fran Saborido-Rey, Taewoo Ryu, Craig Michell, Timothy Ravasi, Xabier Irigoien

[1]King Abdullah University of Science and Technology (KAUST), Division of Biological and Environmental Science & Engineering, Red Sea Research Center, Thuwal 23955-6900, Saudi Arabia
[2]Institute of Marine Research (IIM-CSIC), 36208 Vigo, Spain

[3]KAUST Environmental Epigenetic Program (KEEP), Division of Biological and Environmental Sciences & Engineering, Division of Applied Mathematics and Computer Sciences, King Abdullah University of Science and Technology, Thuwal 23955-6900, Kingdom of Saudi Arabia.
[4]Current address: APEC Climate Center, Busan 48058, South Korea

* Corresponding author: Laura Casas lalaucas@gmail.com

Supplementary information includes nine tables and nine figures.

Supplementary tables S1 to S8 are included in a separate single Excel spreadsheet. Table S1 shows the summary of the transcriptome sequencing and assembly for *Amphiprion bicinctus*. Table S2 is a list the successfully annotated contigs showing correlation and differential expression in the brain during sex change. Table S3 is a list of successfully annotated contigs showing correlation and differential expression in gonads during sex change. Table S4 presents a summary of the Gene Ontology (GO) enrichment analysis in brains of non-transitional stages undergoing sex change. Table S5 presents a summary of the GO enrichment analysis in gonads of male stages undergoing sex change. Table S6 presents a summary of the GO enrichment analysis in gonads of female stages undergoing sex change. Table S7 presents an overview of individual data used in the experiment. Finally table S8 summarizes the gene symbol, forward and reverse primer sequences designed for the RT-qPCR.

The supplementary figures S1 to S10 are shown below.

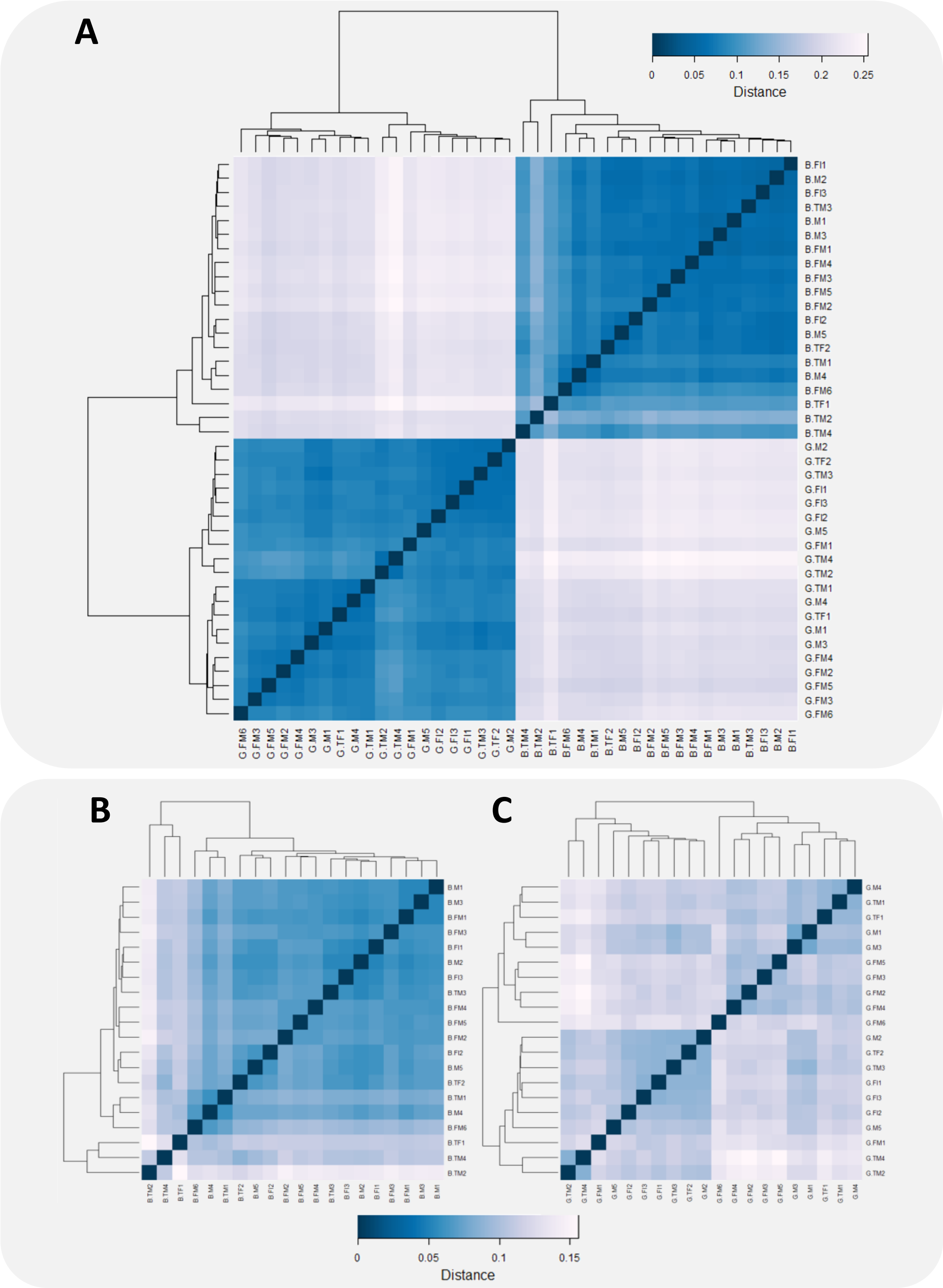

Figure S1. Hierarchical clustering of sample-to-sample distances of the gene expression in clownfish (*Amphiphrion bicinctus*). The heatmaps show different expression between gonad and brain (A), within tissues, with a less distinct pattern in the brain (B) than in the gonads (C).

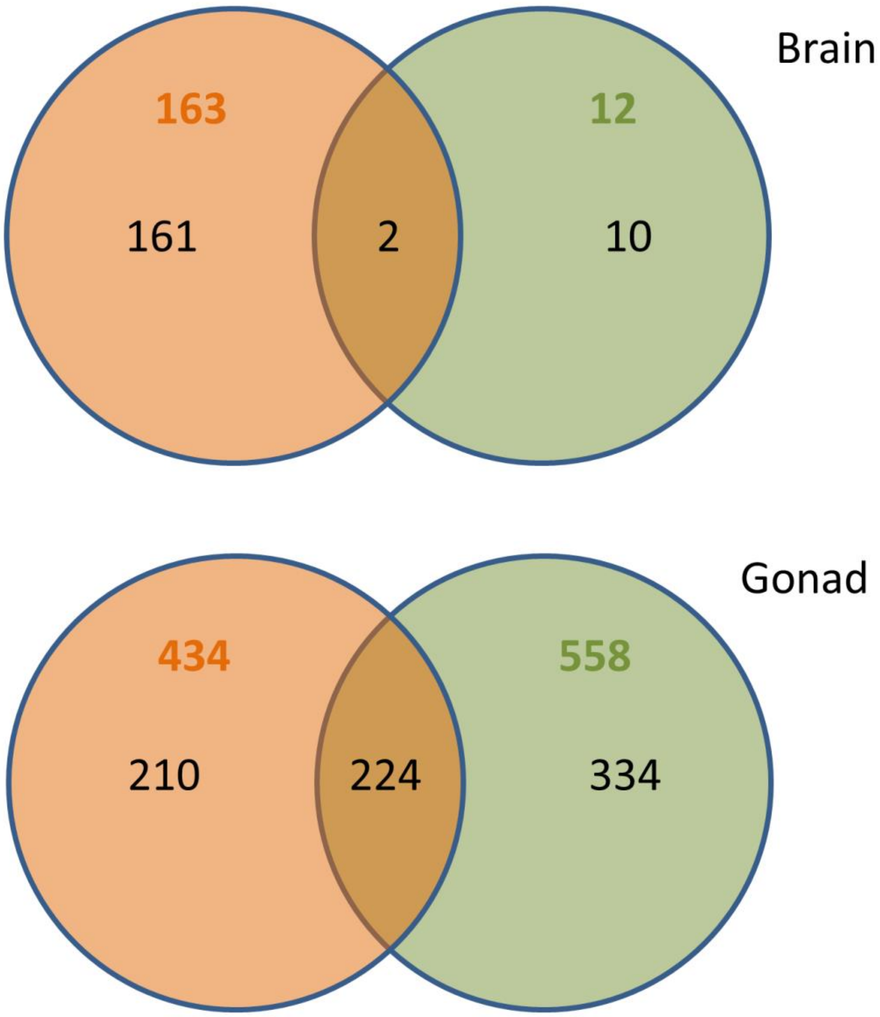

Figure S2. Selected contigs based on two criteria: i) significantly correlated ($p<0.001$, green circle); and ii) significantly different ($p<0.001$) in the pairwise comparisons (brown circle)

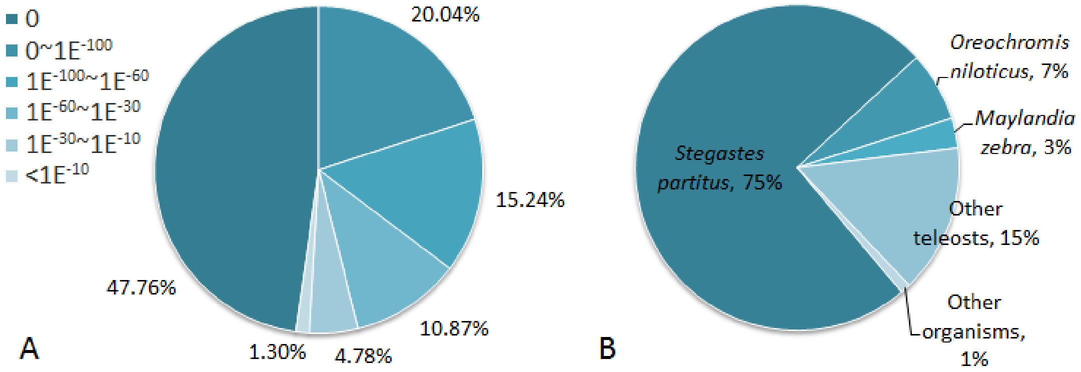

Figure S3. Overview of *A. bicinctus* transcriptome functional annotation A) E-value distribution of transcriptome unigenes. Distribution of e-value for top hits obtained by blast searches against the NCBI nr protein database revealed that 98.7% of the mapped sequences show significant homology (less than 1.0E-10); **B) Annotation coverage by species for the selected contigs based on correlation and/or differential expression during sex change in *A. bicinctus*.** Of the significant selected contigs, 99% had blast hits to teleosts and only 1% corresponded to other organisms. *Stegastes partitus* was the top blast species with 75% of annotation, while 7% corresponded to *Oreochromis niloticus*.

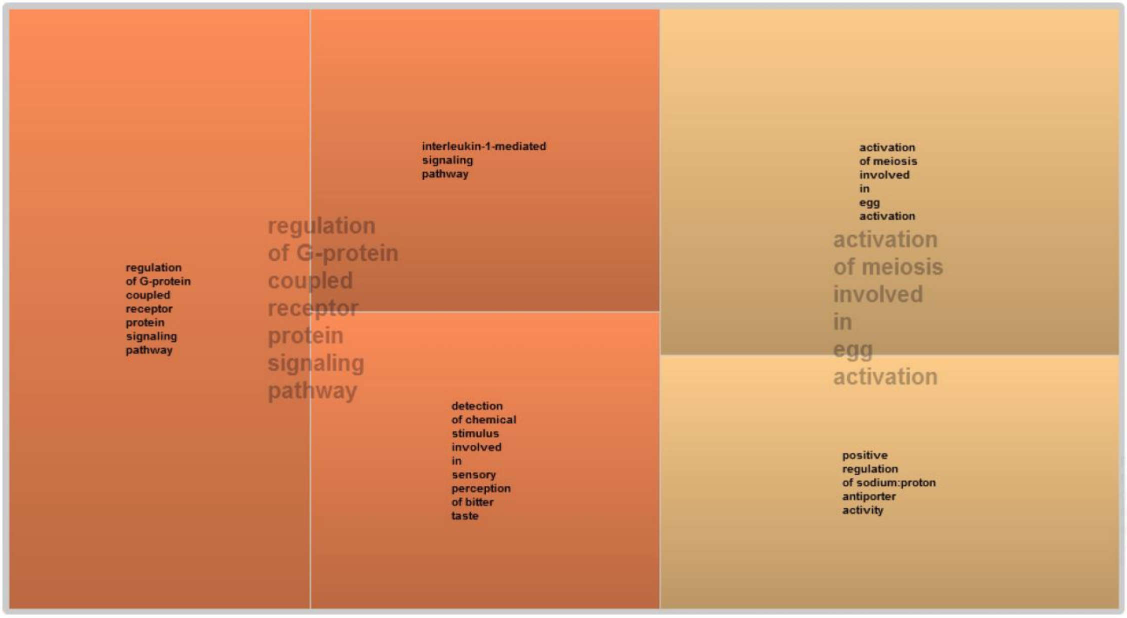

Figure S4. Gene Ontology TreeMap for the genes up-regulated in non-transitional stages in the brain. The box size correlates with the –log10 p-value of the GO-term enrichment. Boxes with the same colour can be grouped together and correspond to the same upper-hierarchy GO-term, which is found in the middle of each box.

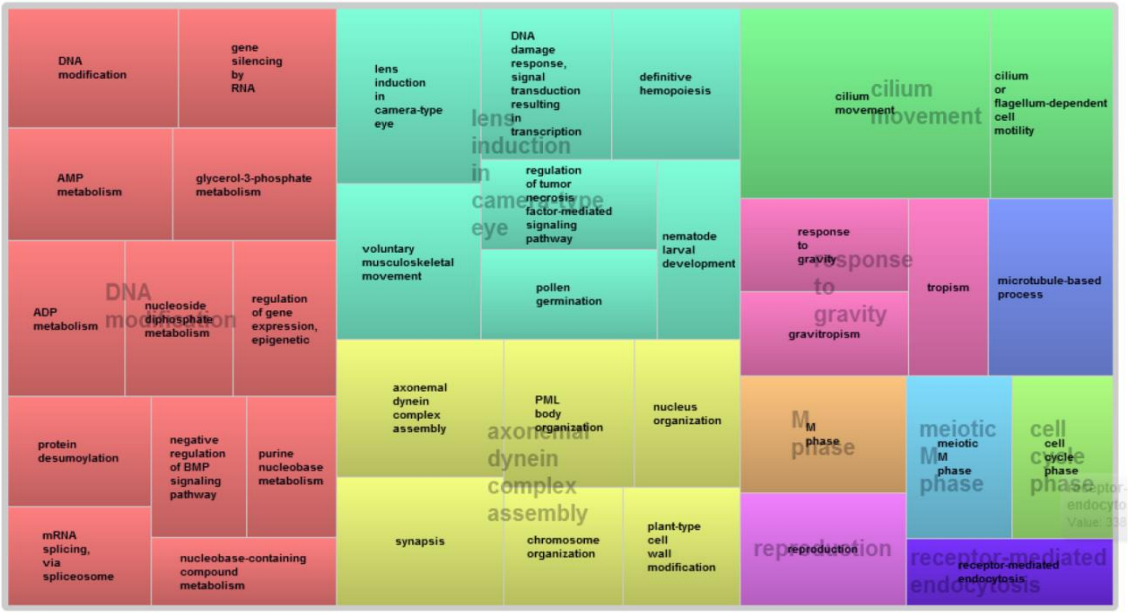

Figure S5. Gene Ontology TreeMap for the 'male correlated' genes in gonads. The box size correlates with the –log10 p-value of the GO-term enrichment. Boxes with the same colour can be grouped together and correspond to the same upper-hierarchy GO-term, which is found in the middle of each box.

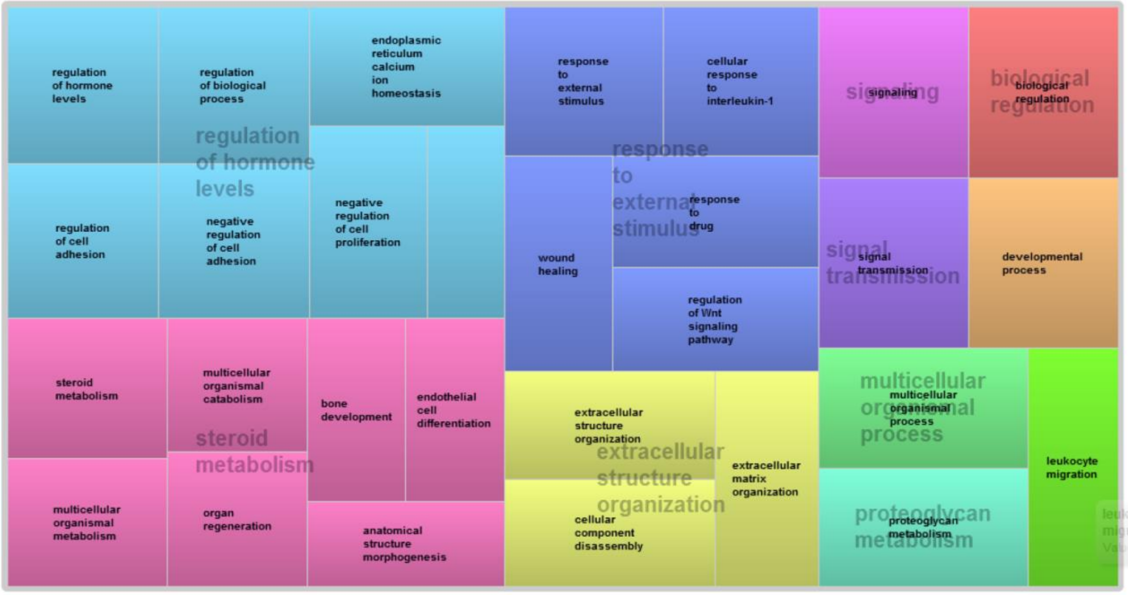

Figure S6. Gene Ontology TreeMap for the 'female correlated' genes in gonads. The box size correlates with the –log10 p-value of the GO-term enrichment. Boxes with the same colour can be grouped together and correspond to the same upper-hierarchy GO-term, which is found in the middle of each box.

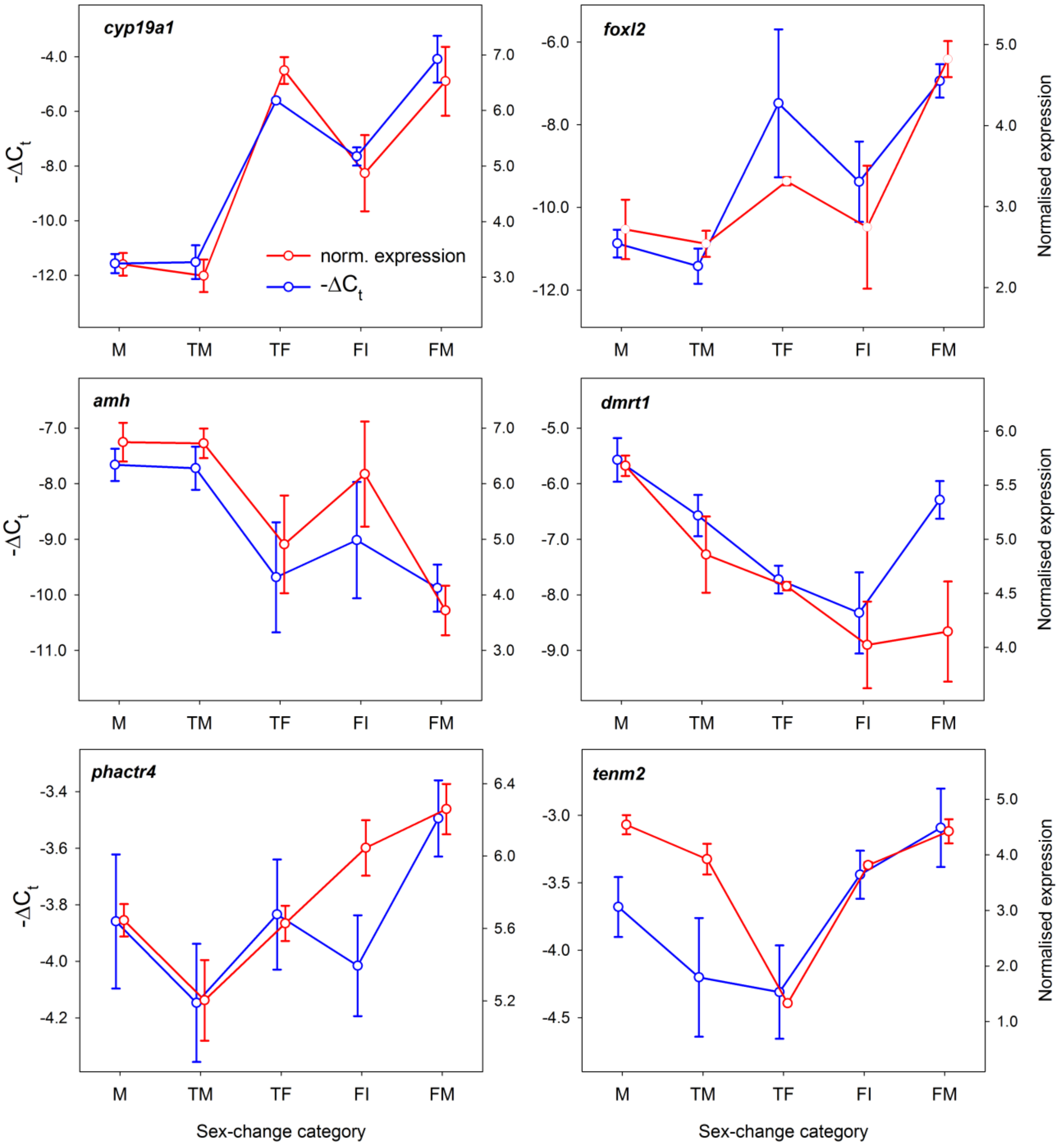

Figure S7. Expression patterns of RNA-seq and qPCR show consistent results for selected genes during sex-change stages. The vertical left axis shows the Delta Ct value obtained through RT-qPCR (blue line) while the vertical right axis shows the normalized expression values (red line) calculated with DEseq. Bars represent the mean for each sex category ± SD.

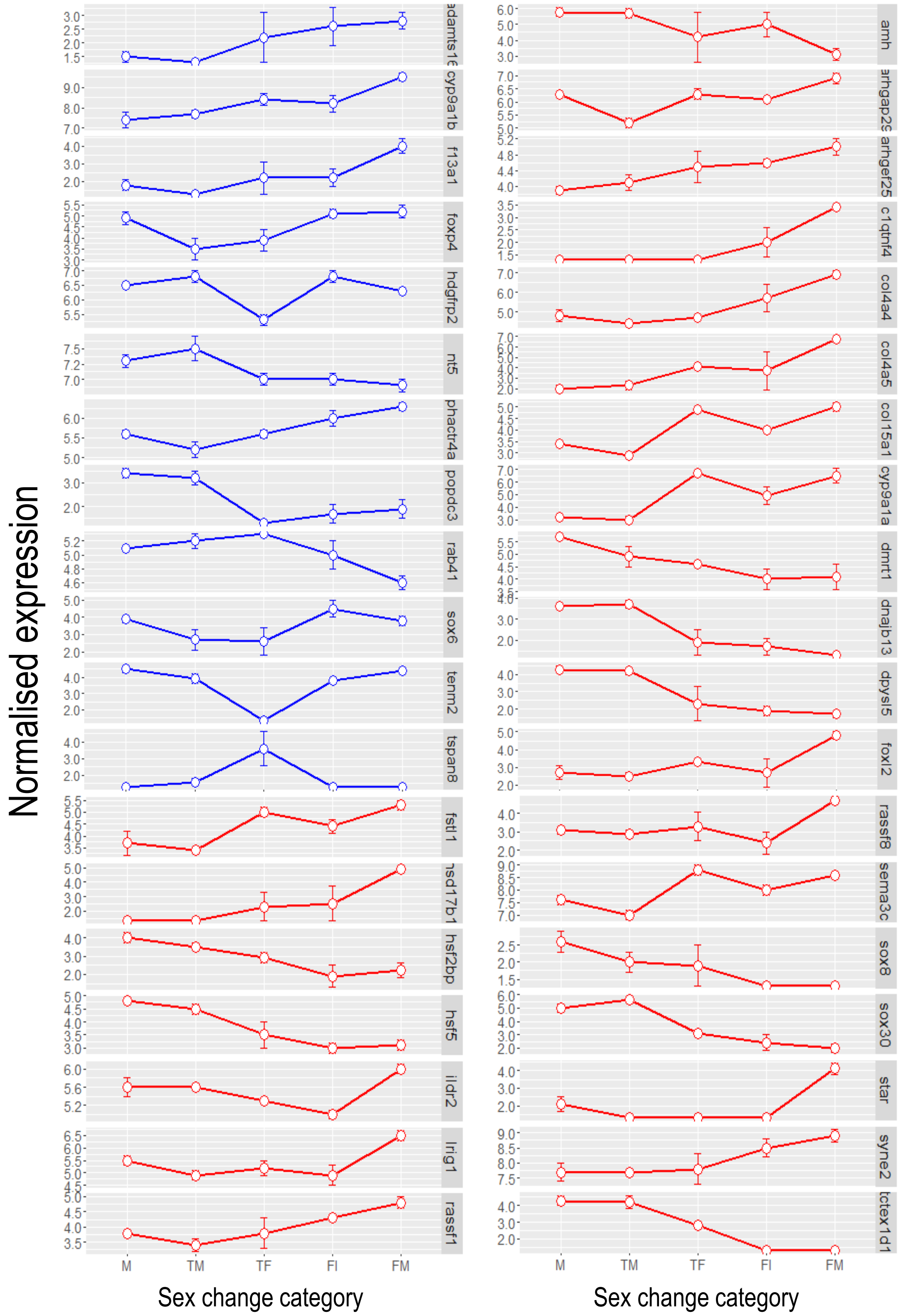

Figure S8. Expression of the discussed 38 genes during sex-change stages in brain (blue) and gonad (red) in clownfish (*Amphiphrion bicinctus*). The vertical axis shows the normalized gene expression levels, bars represent the mean for each sex category ± SE. The description of each gene is found in Table 3, and the associated statistics in supplementary tables S2 and S3. Labels: M – males; TM – transitional males; TF – transitional females; FI– immature females; and FM – mature females.

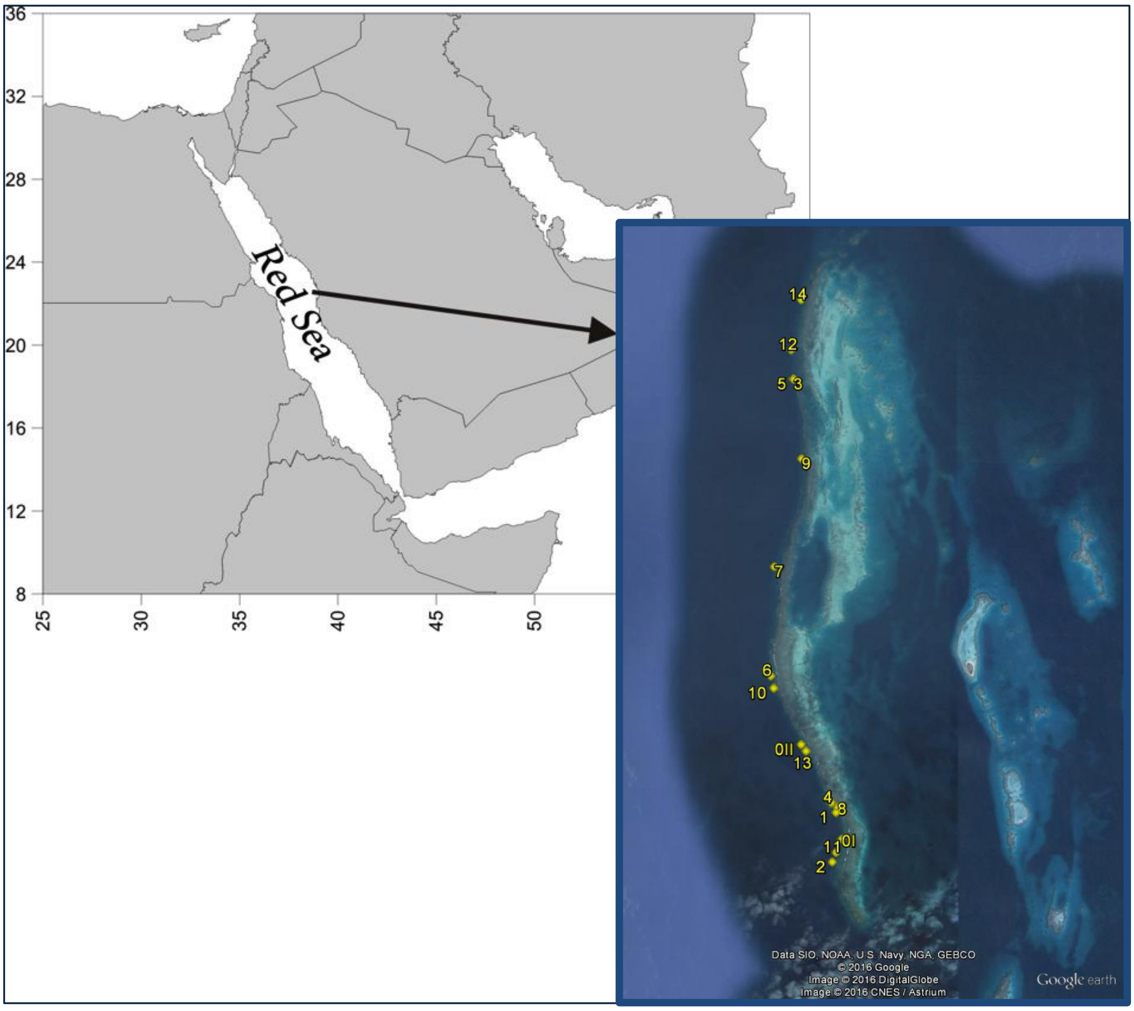

Figure S9. Map showing the geographic location of the 16 clownfish (*Amphiphrion bicinctus*) families analyzed in the present study. MapViewer *6.0 (Golden Software, LLC)* was used to create the map and the satellite image is from Google Earth.

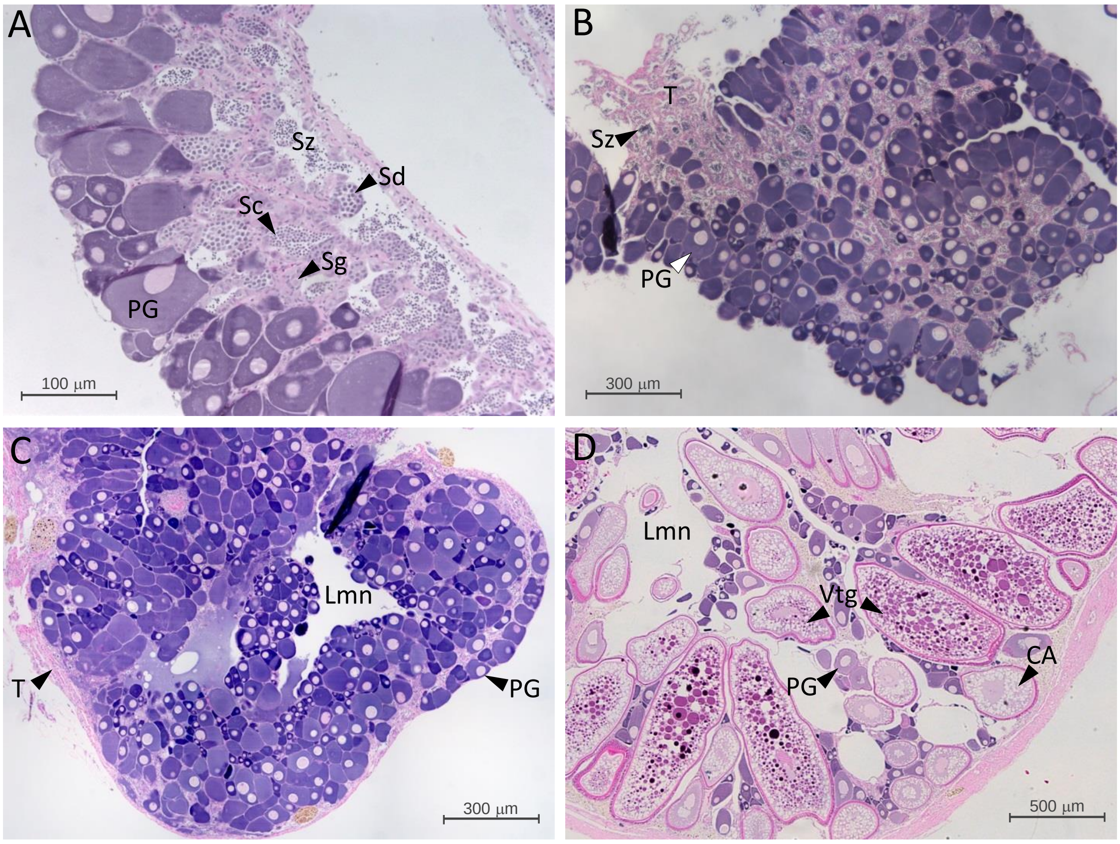

Figure S10. Histological sections of clownfish (*Amphiphrion bicinctus*) ovotestis: A) longitudinal section of a functional male. Ovary and testes occupy half of the gonad each, oocytes are in primary growth stage (PG), while male germ cells are in various stages of development (Sg: Spermatogonia; Sd: Spermatida; Sc: Spermatocyte; Sz: Spermatozoid); B) Transverse section of a transitional fish. Testicular tissue (T) shrinks and mostly spermatozoids are visible, while other male germ cells are degenerated. Ovary tissue is more prominent and only PG oocytes are present. C) Transvers section of an immature female. Testicular tissue (T) is restricted to a small portion in the periphery of the gonad, it is highly degenerated and no germ cells are detected. The lumen of the ovary (Lmn) is visible, but still only PG oocytes are present. D) Transverse section of a mature female. Oocytes in secondary growth stages are found (CA: cortical alveoli; Vtg: vitellogenic oocyte).
